# Laminated Structural Engineering Strategy toward Carbon Nanotube-Based Aerogel Films

**DOI:** 10.1021/acsnano.2c02193

**Published:** 2022-05-19

**Authors:** Chen Fu, Zhizhi Sheng, Xuetong Zhang

**Affiliations:** † Suzhou Institute of Nano-tech and Nano-bionics, Chinese Academy of Sciences, Suzhou 215123, P. R. China; ‡ Division of Surgery & Interventional Science, University College London, London NW3 2PF, United Kingdom

**Keywords:** carbon nanotubes, Kevlar nanofibers, aerogel film, EMI shielding, Joule heating

## Abstract

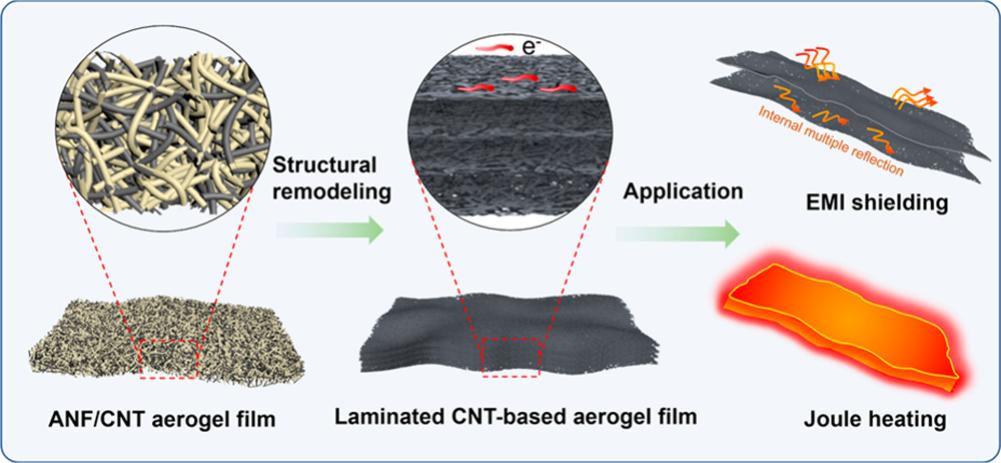

Aerogel films with a low density are ideal candidates to meet lightweight application and have already been used in a myriad of fields; however, their structural design for performance enhancement remains elusive. Herein, we put forward a laminated structural engineering strategy to prepare a free-standing carbon nanotube (CNT)-based aerogel film with a densified laminated porous structure. By directional densification and carbonization, the three-dimensional network of one-dimensional nanostructures in the aramid nanofiber/carbon nanotube (ANF/CNT) hybrid aerogel film can be reconstructed to a laminated porous structure with preferential orientation and consecutively conductive pathways, resulting in a large specific surface area (341.9 m^2^/g) and high electrical conductivity (8540 S/m). Benefiting from the laminated porous structure and high electrical conductivity, the absolute specific shielding effectiveness (SSE/*t*) of a CNT-based aerogel film can reach 200647.9 dB cm^2^/g, which shows the highest value among the reported aerogel-based materials. The laminated CNT-based aerogel films with an adjustable wetting property also exhibit exceptional Joule heating performance. This work provides a structural engineering strategy for aerogel films with enhanced electric conductivity for lightweight applications, such as EMI shielding and wearable heating.

Aerogel, a three-dimensional porous structure exhibiting a low density and high specific surface area, is an ideal candidate to meet the requirements of electronic and energy devices for lightweight applications.^1−4^ Since the finding of silica aerogel, various aerogels have been widely studied, such as polymeric aerogel,^5,6^ nanocellulose aerogel,^7,8^ aramid nanofiber (ANF) aerogel,^9,10^ cellulose nanofibrils aerogel,^11^ graphene aerogel,^12−14^ carbon nanotube aerogel,^15^ MXene aerogel,^16,17^ and their composite aerogels.^18,19^ The diversity of aerogel types promises them different properties, and these aerogels have already been widely used in the fields of electrical,^20,21^ optical,^22,23^ and catalysis.^24,25^ In recent years, in addition to focusing on the assembled building blocks of aerogels, the microstructure design for the target aerogels has also attracted more and more attention.^26^ Some studies suggest that the microstructure of the aerogel has a crucial effect on its properties. For example, with a directional alignment structure, graphene aerogel has excellent mechanical and thermal conductivity, in comparison with the graphene aerogel possessing randomly distributed pores.^27^ By introducing a layered structure, the resulting nanocrystal cellulose aerogel shows a chiral pressure response phenomenon.^28^ Carbon nanofiber aerogels with hierarchical structures exhibit excellent elasticity and fatigue resistance in the range from −100 to +300 °C.^29^ By virtue of the anisotropic structure of natural wood, cellulose nanofiber aerogel with a layered structure has excellent compressive performance.^30^ Hence, the design and preparation of aerogels with special microstructure have important implications for their performance enhancement.

However, with the diversification of application requirements and the complexity of scenarios, the aerogel needs to be designed in various forms besides bulk style, especially a self-supporting aerogel film.^31−34^ As a pioneer in aerogel, silica aerogel films were first studied and have been used for intermetallic dielectrics and heat insulation due to their low dielectric constant and low thermal conductivity.^35,36^ With the development of varieties of aerogels, multitudinous aerogel films with different functions were prepared, such as conductive aerogel films^37,38^ and insulating polymeric aerogel films.^23,31,39^ Among these, electrically conductive aerogel films have important application prospects in the field of supercapacitors,^40^ lithium batteries,^41^ and electromagnetic protection.^38^ Carbon nanotube (CNT), feigned as a cylindrical graphene sheet, with excellent electrical conductivity,^42^ is an ideal construction unit for conductive aerogel film. However, the random three-dimensional porous structures of aerogel go against the construction of a conductive network for CNTs, resulting in dissatisfactory electrical conductivity.^43^ Some studies show that densification technology can improve the electrical conductivity and mechanical properties of CNTs film.^44,45^ Inspired by the microstructural design of bulk aerogel, the introduction of densification technology into the carbon nanotube aerogel film may be an effective way to improve the conductivity and mechanical properties of the carbon-based aerogel film. Recently, the hydrogel can be prepared into a film by hot-pressing. During the hot-pressing, the hydrogels are dried under atmospheric pressure, which causes the collapse of the pore structure.^46^ Therefore, the challenge remains in constructing a CNT-based aerogel film with both high conductivity and high porosity.

In this work, we introduce a laminated structural engineering strategy for the establishment of a self-supporting CNT-based aerogel film with a densified layer and hierarchical porous structure, through the densification technology and carbonization process. The densified laminated structure can effectively improve the electrical conductivity and mechanical strength of the final constructs. The electrical conductivity of the CNT-based aerogel film can be up to 8540 S/m. The carbonization treatment can effectively create microporous structures. The combination of densified laminated structure and porous structure endows the CNT-based aerogel film with a high electromagnetic interference (EMI) shielding performance. The resulting CNT-based aerogel film exhibited excellent mechanical properties with a specific tensile strength of 117.6 MPa g^–1^ cm^3^. Benefiting from the high electrical conductivity, the laminated CNTs aerogel film also shows exceptional Joule heating performance. This work provides guidance for designing advanced aerogel films with enhanced electric conductivity for lightweight electronic device applications, such as EMI shielding and wearable heating.

## Results and Discussion

### Fabrication and Characterization of CNT-Based Aerogel Film

**Scheme 1:**
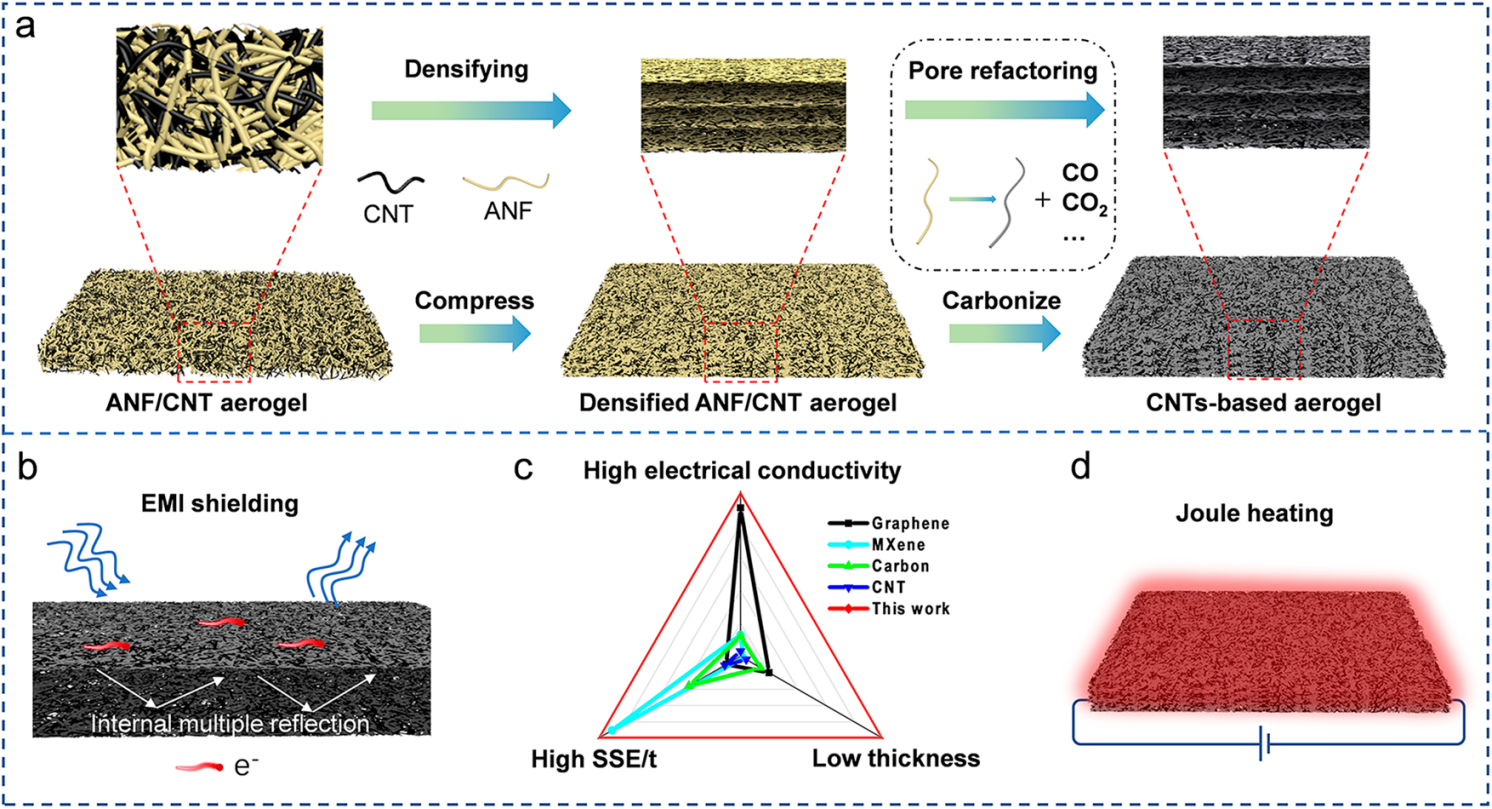
Schematic Illustration of the Fabrication Procedure and Features of the CNT-Based Aerogel Film (a) ANF/CNT aerogel film can be formed with a three-dimensional fiber network. By directional compressing, the lamination structure can be generated. Following carbonization, CNT-based aerogel film with a dense fiber layer and laminated structure can be obtained as well as pore refactoring in between the layers. (b) Schematic illustration of EMI shielding behavior of the CNT-based aerogel film. (c) Radical plot comparing the thickness, density, and SSE/*t* for CNT-based aerogel film and other aerogel materials including graphene, CNT, and MXene. (d) Schematic illustration of Joule heating performance of the CNT-based aerogel film.

A three-step laminated structural engineering strategy was designed to prepare CNT-based aerogel films as illustrated in Scheme 1. In our previous work, ANFs can be dimensionally matched and assembled with CNTs, which can form a double network aerogel film with high conductivity and mechanical strength.^37^ On the basis of ANF/CNT aerogel film, we provide a strategy to obtain a laminated CNT-based aerogel film with excellent electrical conductivity, much lower density, and far thinner thickness. First, we prepared the ANF/CNT hydrogel film through the sol–gel transition of ANF dispersion mixed with CNTs (10:2, mass ratio). Then, the ANF/CNT aerogel film was obtained by freeze-drying of the ANF/CNT hydrogel film. Finally, by directionally compressing, ANFs and CNTs were densely arranged in the horizontal plane, which is beneficial for forming a better conductive pathway. In the progress of densification, the three-dimensional nanofiber network can be densified to form a laminated structure. For the sake of enhanced conductivity, the densified ANF/CNT aerogel film was dealt with carbonization at different temperatures under the protection of argon. During carbonization, gases with a small molecular weight (such as CO, CO_2_) are generated by decomposing Kevlar nanofibers, which is beneficial for the formation of micropores. By combining a porous structure with a dense conductive layer, the such CNT-based aerogel film shows high electrical conductivity as well as low density and low thickness, resulting in excellent EMI shielding and Joule heating performance. Compared with other aerogel materials based on graphene, MXene, carbon, and CNT, such CNT-based aerogel films show the highest SSE/*t* performance with high electrical conductivity and low thickness (Scheme 1c).

**Figure 1:**
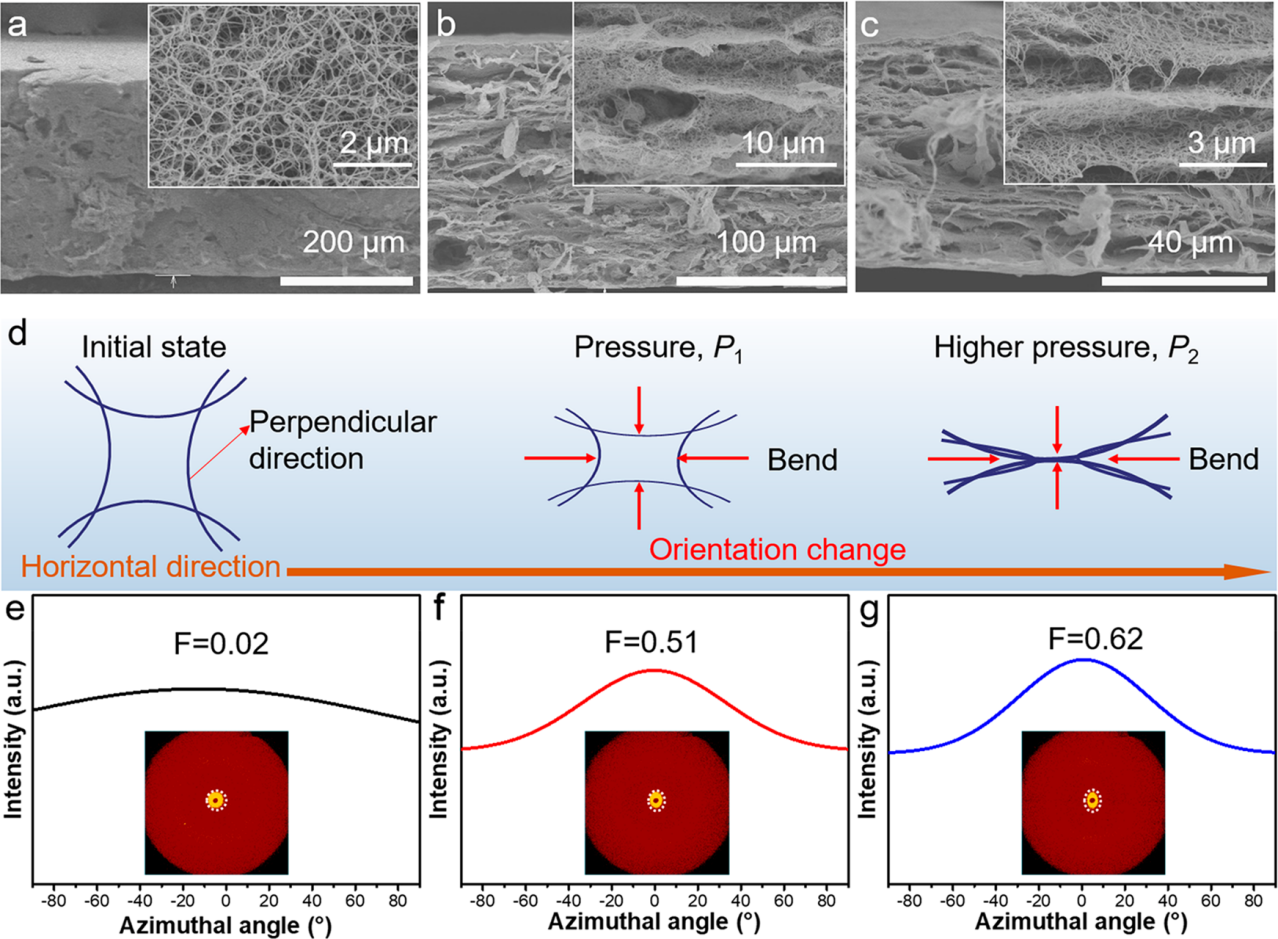
Structural evolution of ANF/CNT aerogel films made by the laminated structural engineering strategy. SEM images of ANF/CNT aerogel films (a) before and after different compressive strains: (b) 10 MPa and (c) 20 MPa; the inset images are their corresponding magnified SEM images. (d) Schematic representation of ANF/CNT aerogel films under different pressures. The azimuthal plots are in the range from −90° to +90° under different compressive strains: (e) 0 MPa, (f) 10 MPa, and (g) 20 MPa, and the inset images are the corresponding diffraction patterns.

The formation process of densified laminated structure from the three-dimensional nanofiber network was studied by scanning electron microscopy (SEM) and wide angle X-ray scattering (WAXS). Without a directional compression (initial state), the ANFs and CNTs are randomly orientated. As shown in Figure 1a, ANFs are interconnected to form a three-dimensional porous network and CNTs are intertwined and randomly distributed throughout the whole nanofibrous network, creating a continuous efficient conductive network. Since ANFs and CNTs can be dimensionally matched, they are assembled and twined with each other, inducing CNTs to be evenly dispersed in the network of the ANFs. Due to the assembly of ANFs through hydrogen bonding in the gel progress, the ANFs are cross-linked rather than independent. Under compression, the ANFs and CNTs with unhorizontal-layout in the three-dimensional network are orientated to horizontal, causing structural evolution, as illustrated in Figure 1d.^47^ A highly orientated structure can be observed after compressive treatment in Figure 1b,c. We used WAXS to evaluate the orientation performance of ANFs and CNTs with the compression process. After directional densification, we can observe the (002) reflection of CNTs and marked anisotropy in the WAXS patterns. The radial intensity profile of D-ANF/CNT displayed a broad peak at 1.5 Å^–1^, which is attributed to the highly imperfect packing of CNTs (Figure S1).^48^ From Figure 1e–g, we can obviously observe the orientation structure after compression. Then, we used Herman’s orientation factor (*f*), which is used to analyze the alignment of polymer, to evaluate the orientation of ANFs and CNTs in the film. The *f* values from 0 to 1 mean a random distribution to a completely oriented distribution.^49^ The *f* value of the original aerogel is almost close to 0 (Figure 1e), which means the random distribution of ANFs and CNTs. By compressing with 50% and 80% strain, the *f* values are increased to 0.51 (Figure 1f) and 0.62 (Figure 1g), respectively, representing the preferential orientation of ANFs and CNTs in the film. This interesting structural evolution process is also confirmed by calculating the orientation factor (OF) (Figure S2) from the fast Fourier transform (FFT) of SEM images.^50^ Note that OF = 0.5 means that the nanotubes are randomly oriented and OF = 1.0 is perfectly aligned. UP to 80% strain, the randomly oriented ANFs and CNTs are deformed into the orientation normal to the compression direction, and the OF is steadily increased from 0.62 to 0.74. This densified structure can enhance the mechanical strength.

**Figure 2:**
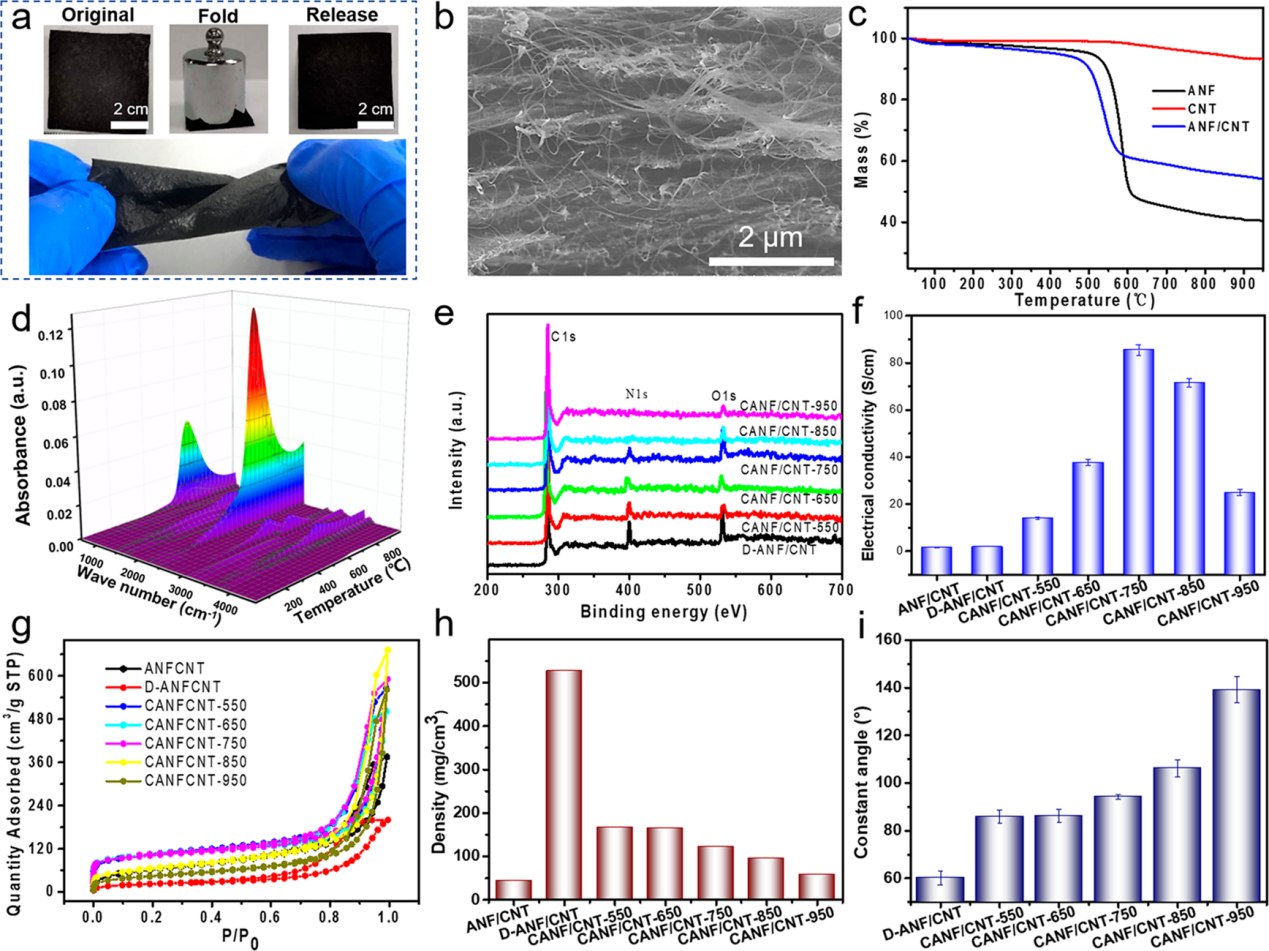
Characterizations and properties of CNT-based aerogel films. (a) Photographs of the folding, releasing, and twisting process of CANF/CNT-750 aerogel film. (b) Cross-section SEM images of CANF/CNT-750 aerogel film. (c) TG curves of pure CNT, ANF aerogel film, and ANF/CNT aerogel film. (d) Thermal decomposition infrared spectroscopy of D-ANF/CNT. (e) XPS spectra of the CNT-based aerogel films with different carbonization temperatures. (f) Electrical conductivity of ANF/CNT aerogel film, D-ACNF/CNT film, and CNT-based aerogel films carbonized at different temperatures. (g) Nitrogen adsorption–desorption isotherm of CNT-based aerogel films. (h) Density of CNT-based aerogel films carbonized at different temperatures. (i) Static water contact angle of CNT-based aerogel films carbonized at different temperatures.

Although the densification progress can bring a laminated structure, the microporous structure, as well as the mesoporous structure, of the resulting aerogel film might disappear, resulting in an increased density of the film. Then, we introduce the carbonization progress to achieve the regeneration of the microspores. Benefiting from the excellent toughening of CNTs,^51,52^ the as-prepared densified and carbonized CNT-based aerogel films are highly flexible and can be rolled up and even folded without any damage (Figure 2a). As the mechanical performance is a very important factor for a film to use in practice, the mechanical properties of the CNT-based aerogel films with different carbonization temperatures were investigated, as shown in Figure S3. With densification, D-CANF/CNT shows great enhanced mechanical properties compared with ANF/CNT. The tensile strength increased from 0.3 to 31.6 MPa (Figure S3). After carbonization, the mechanical properties of CANFCNT are reduced, due to the decomposition of ANF. For CANF/CNT-550, the film has a better strain ability compared with other films with a higher carbonization temperature, because of the incomplete pyrolysis of ANFs at 550 °C. The tensile strength of CANF/CNT-550 is above 20 MPa, and its specific strength can be up to 117.6 MPa g^–1^ cm^3^, which exhibits excellent mechanical strength in comparison with metal and other carbon-based materials.^46^ As the carbonization temperature rises, the tensile strength of CNT-based aerogel film decreases from 20 to 12 MPa, due to the complete pyrolysis of ANFs.

The morphology and microstructure of CNT-based aerogel film were observed by SEM images, as shown in Figure 2b. We can clearly observe the laminated structure, which consists of carbonized ANFs and CNTs. This inerratic layer-like structure is inherited after the carbonization progress, which means the pyrolysis of ANFs cannot change the laminated structure of densified aerogel films. The CNT-based aerogel films exhibit a smooth surface structure (Figure S4). During the gelling process, due to the rapid solution exchange on the surface, a compact skin-like structure will be formed on the surface of the ANF hydrogel film. Some holes can be observed on the surface of CNT-based aerogel film, which may be caused by the carbonization process with the generation of small molecule gas during the carbonization progress. The orientation analysis of such CNT-based aerogel film was also conducted. As shown in Figure S5, the CANF/CNT aerogel films with different annealing temperatures have similar microstructures and orientation factors, which demonstrate that the annealing process has no obvious effect on the biaxial orientation of CNTs and carbonized ANF.

In order to investigate the carbonization process of densified CNT-based aerogel film. We first studied the pyrolysis progress of the densified ANF/CNT aerogel film by a thermogravimetry (TG) test. As shown in Figure 2c, with the temperature increase, the densified ANF/CNT aerogel film shows three weight loss stages. Before 300 °C, 6.1% weight loss was caused by the desorption of water molecules in the film. As the temperature further rises up to 600 °C, the film has a significant weight loss of 32.9%. In the temperature range 450–600 °C, the intermolecular and intramolecular hydrogen bonds were broken, and the amide bond of ANFs starts to break, along with the generation of ammonia, carbon dioxide, and other gases.^53^ Those gases contribute to the formation of micropores, which can be proven by the results of the thermogravimetry-Fourier transform infrared (TG-FTIR) test. Because of those micropores, the density of the CNT-based aerogel film is greatly reduced from 0.53 to 0.16 g/cm^3^. After 600 °C, the weight of the film declines slowly, owing to the further carbonization of Kevlar nanofibers. The above results will guide us to prepare CNT-based aerogel films under different temperatures. According to the TG results of ANF aerogel film, pure CNT, and ANF/CNT aerogel film, we also can calculate the mass ratio of CNTs in the CANF/CNT aerogel film. For CANF/CNT-750, the mass ratio of CNT in the aerogel film is 74.36%. For pure ANF aerogel film, it still maintains the three-dimensional fiber network structure after carbonization progress, as shown in Figure S6.

Then, the TG-FTIR was used to investigate the gases generated in the progress of decomposition. As shown in Figure 2d, before 300 °C, there are few gases generated, almost donated by H_2_O. When the temperature is raised above 300 °C, the characteristic peaks of various gases can be observed on the Fourier transform infrared (FTIR) spectrum. It is worth noting that, following the increase in temperature, the CO_2_ gradually becomes the dominant component of the gases generated by the decomposition of Kevlar, and its maximum amount is reached at 638 °C. Figure S7 is the FTIR spectrum of decomposed gases at 638 °C, and the characteristic peaks of H_2_O, CH_4_, CO_2_, CO, and NO_2_ can be observed.^54^ During the decomposition of these gases, a large number of micropores are recreated in the film, causing a high specific surface area.

According to the results of TG analyses, we prepared several CNT-based aerogel films with different carbonization degrees under different carbonization temperatures (550, 650, 750, 850, and 950 °C), denoted as CANF/CNT-550, CANF/CNT-650, CANF/CNT-750, CANF/CNT-850, and CANF/CNT-950, respectively. As mentioned above, treated by different carbonization temperatures, the ANFs have different decomposition degrees, manifested in the changes of element contents, which can be identified by the results of X-ray photoelectron spectroscopy. As shown in Figure 2f, compared with D-ANF/CNT, the nitrogen content of the CANF/CNT aerogel films was reduced with increasing the carbonization temperatures. Especially, the peak of the nitrogen element disappeared in the curves of CANF/CNT-950, which means the amide bond of ANFs was broken and the nitrogen atom came off completely. For CANF/CNT-550, CANF/CNT-650, and CANF/CNT-750, the oxygen element content is increased (Figure S8), which is contributed to the generation of CH_4_. CH_4_ and CO_2_ were generated in the carbonization progress (Figure S9). The generation of CH_4_ and CO_2_ reaches their maximum at 680 and 640 °C, respectively.

Benefiting from the densified structure and carbonization progress, the densified laminated CNT-based aerogel films show excellent electrical conductivity (Figure 2f). During the densified process, the CNTs are closely packed, which is beneficial for electron transport, resulting in more efficient conductive networks. The carbonization progress converts the poorly conducting polymer into a conductive carbon material, which also improves the electrical conductivity. As the carbonization temperature rises from room temperature to 750 °C, the CNT-based aerogel films show an increased electrical conductivity. As a common method to investigate the degree of carbonization, Raman spectra of CNT-based aerogel films were investigated, as shown in Figure S10. Compared with densified ANF/CNT aerogel film, the CNT-based aerogel films show stronger peaks at 1584.6 and 1608.4 cm^–1^, assigned to the in-plane vibration of the C–C bond (G) and C–C ring stretching, respectively.^55^ The peak at 1335 cm^–1^ (D-band) almost disappears for the CANF/CNT-950 aerogel film. As is well-known, the D-band is caused by a disorder or defects. The crystal phase compositions of CNT-based aerogel films were also studied by XRD (Figure S11). Without carbonization, the densified ANF/CNT aerogel film has a broad peak at 22.8°, assigned to (200) reflections of ANF.^56^ After carbonization, there are no obvious peaks at 22.8° for all CNT-based aerogel films, signifying the carbonization degree of ANFs. However, the electrical conductivity of CNT-based aerogel films was not enhanced continuously with the carbonization temperature rising. CANF/CNT-750 has the highest electrical conductivity among the CNT-based aerogel films, and its conductivity is about 8540 S/m (Figure 2e), which is much higher than those of other CNT-based aerogels.^15,47,57^ As mentioned above, during the carbonization progress, small molecule gases will be generated during the carbonization of Kevlar, resulting in a more porous structure, which is a disadvantage for electrical conductivity, although the higher carbonization temperature can contribute to a better carbonization degree. Therefore, to obtain laminated porous CNT-based aerogel films with excellent electrical conductivity, the trade-off between carbonization degree and porous structure should be taken into consideration.

In order to investigate the porous structure formation of CNT-based aerogel films in the carbonization progress, we used a nitrogen adsorption test to study its specific surface area and pore size distribution. As shown in Figure 2g, the isotherm of all samples exhibits a rapid increase in the low-pressure range and a hysteresis loop, indicating the existence of micropores and mesopores. The specific surface area and the pore size distribution of CNT-based aerogel films were studied, as illustrated in Figures S12 and S13, respectively. The ANF/CNT aerogel film exhibited a specific surface area of about 223.6 m^2^/g and has two types of micropores with diameters of 0.7 and 0.8 nm. After directional densified compression, the specific surface area of D-ANFCNT just has 77.8 m^2^/g and the micropores are almost disappeared. After the carbonization progress, the pyrolysis of ANFs and the generated gases endows the CANF/CNT-550 aerogel film with a high specific surface area of 341.9 cm^2^/g, even higher than that of the undensified ANF/CNT aerogel film. From the corresponding pore size distribution curve, we can see the amount of micropores with a diameter of 0.6 nm generated. When the carbonization temperature increased to 650 and 750 °C, the specific surfaces of the CANF/CNT-650 aerogel film and CANF/CNT-750 aerogel film slightly reduced to 335.8 and 328.6 m^2^/g, respectively, which are much larger than that of the reported CNT aerogel film (53.0 m^2^/g).^10^ After further increasing the carbonization temperature to 850 °C, or even to 950 °C, the small micropores and mesopores will grow into larger porous structures (Figure S13), which leads to the reduction of the specific surface area. As for CANF/CNT-850 aerogel film and CANF/CNT-950 aerogel film, the micropore with a diameter of 0.6 nm disappears and the specific surface area decreases gradually to 222.6 and 151.3 m^2^/g, respectively.

The influence of different carbonization temperatures on the density of CNT-based aerogel films was also studied (Figure 2h). The density of ANF/CNT aerogel film is closely around 43 mg/cm^3^. After the directional densification, the density of D-ANF/CNT film is greatly increased, due to the dense packing of ANFs and CNTs. By carbonization treatment, the density of the CANF/CNT aerogel films is significantly decreased, because of the pyrolysis of ANFs. As the carbonization temperature increases gradually, the densities of CANF/CNT aerogel films are gradually decreased from 167 mg/cm^3^ (CANF/CNT-550 aerogel film) to 58 mg/cm^3^ (CANF/CNT-950 aerogel film). Interestingly, the pyrolysis of ANFs can not only adjust the density of carbon film but also influence the wetting property of CNT-based aerogel films (Figure 2i and Figure S14). Generally, Kevlar is hydrophilic and CNTs are hydrophobic. Therefore, the wetting property of the CNT-based aerogel film can be adjusted by the carbonization degree of Kevlar. Without carbonization, the D-ANF/CNT film exhibits good hydrophilicity where the static water contact angle (CA) is 60.2°. The CA rises slowly from 85.9° to 106.1° with the carbonization temperature rising from 550 to 850 °C. With the carbonization temperature further increased, the carbon films are hydrophobic. As for CANF/CNT-950, the CA can reach 139.2°. The carbon residue of Kevlar can be used to explain the wetting property of CANF/CNT aerogel films, which have already been proven by the TG results. Due to the reduced proportion of Kevlar in the film, the CNTs will play a leading role in the hydrophobicity of the film. Therefore, the CANF/CNT-950 aerogel film shows satisfactory hydrophobicity (up to 139.2°), contributing to the CNTs.

### EMI Shielding Performance

**Figure 3:**
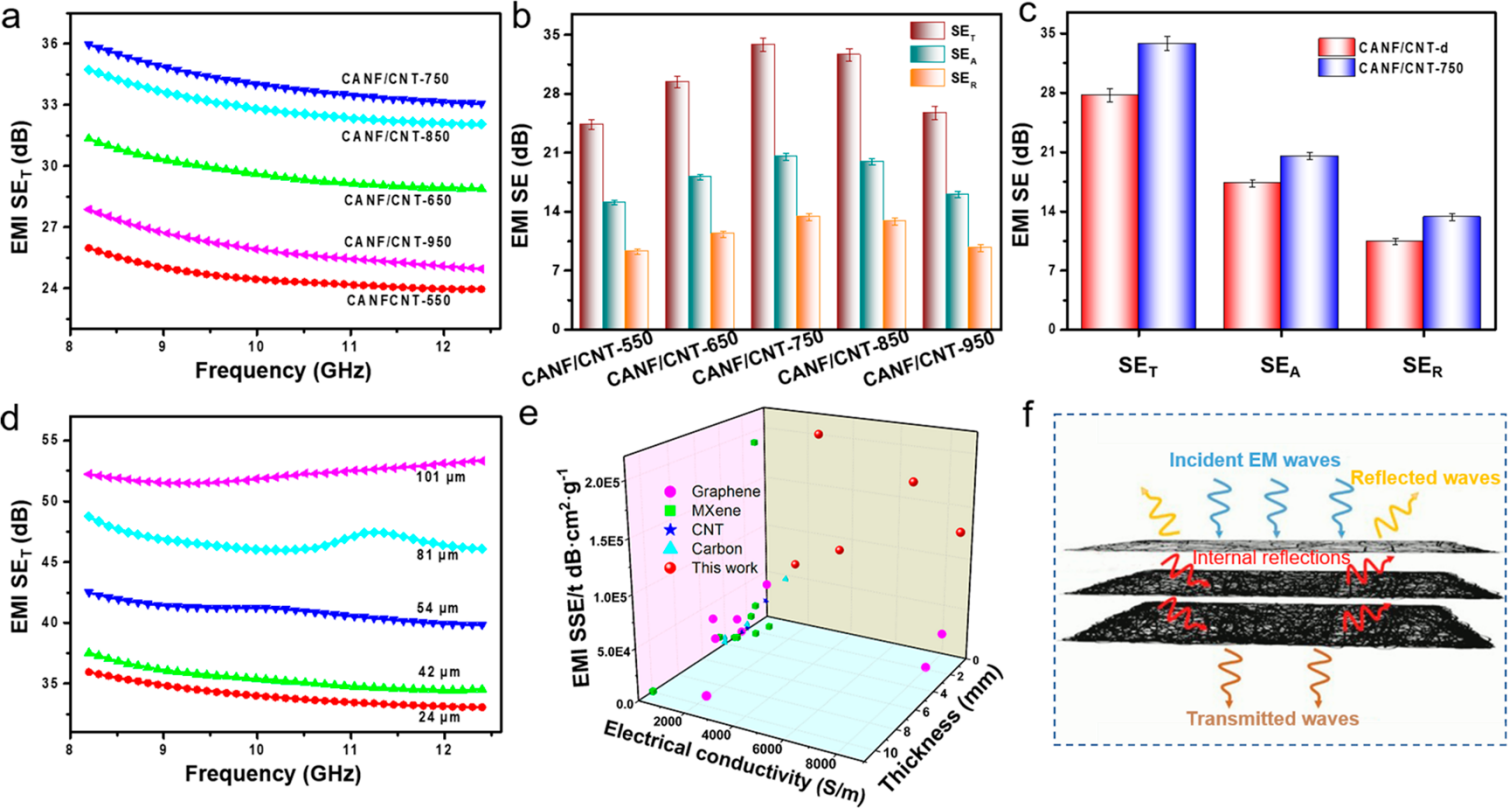
Electromagnetic interference shielding performance of CNT-based aerogel films. (a) EMI SE_T_ of the CNT-based aerogel films. (b) Average EMI SE_T_, EMI SE_A_, and SE_R_ of the CNT-based aerogel films. (c) EMI SE comparison of the CANF/CNT-750 aerogel film and CANF/CNT-d film. (d) EMI SE_T_ of CNT-based aerogel films with different thicknesses. (e) SSE/*t* versus thickness and density of the CNT-based aerogel films compared with those of materials reported in the literature. (f) Proposed EMI shielding mechanism.

The CNT-based aerogel films with a laminated structure and high electrical conductivity are expected to exhibit excellent EMI shielding performance. The EMI shielding effectiveness (SE) of prepared CNT-based aerogel films was measured at a frequency of 8.2–12.4 GHz (X-band) with the waveguide method. According to Simon’s formula^55^
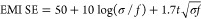
where σ (S/cm) is the electrical conductivity, *f* (MHz) is the frequency of the incident microwave, and *t* (cm) is the thickness of the film. It can be clearly seen that the EMI shielding performance is closely bound up with the electrical conductivity. The values of EMI SE of the CNT-based aerogel films go with its electrical conductivity (Figure 3a). With the highest electrical conductivity, the CANF/CNT-750 aerogel film shows the best EMI shielding performance. However, the effect of microstructure on the EMI shielding performance is ignored by the Simon’s formula. Numerous researches demonstrate that the microstructure plays an important role in the EMI shielding progress.^38,42,48^ For the CANF/CNT-750 aerogel film with a lamellar porous structure, it shows a better electromagnetic shielding performance compared with theoretical results (Figure S15), calculated by the Simon’s formula. The lamellar porous structure induced multiple reflections of incident microwaves, resulting in more propagation paths and polarization loss of incident microwaves. It is worth mentioning that the thickness of CNT-based aerogel films is just about 24 μm, and its density is in the range between 0.06 and 0.17 g/cm^3^, which are lower than those of other nonmetallic materials with such EMI shielding performance.^58^ The outstanding shielding performance of CNT-based aerogel films is attributed to the densified layers and the porous laminated structure, resulting in excellent electrical conductivity and multiple reflections of incident electromagnetic waves, respectively.^59,60^

Typically, the total EMI SE (SE_T_) is contributed by reflection effectiveness (SE_R_), absorption effectiveness (SE_A_), and multiple internal reflection effectiveness (SE_M_).^61,62^ Commonly, at higher EMI SE values (>10 dB), the multiple internal reflections are merged in the absorption, because the rereflected waves by the multiple internal reflections are absorbed or dissipated in the form of heat, which reflect through the reflection effectiveness.^63,64^ As shown in Figure 3b, SE_A_ and SE_R_ display the same trend as SE_T_, related to the electrical conductivity. Obviously, for all CNT-based aerogel films, the SE_A_ values are higher than the SE_R_ values, indicating that absorption has more contribution to EMI shielding than reflection for such a porous laminated carbon film.

As a comparison, we dried the ANF/CNT hydrogel film directly without the solvent replacement of deionized (DI) water and *tert*-butyl alcohol, which can protect the microstructure of hydrogel from breaking or deteriorating, and the obtained carbon film is named CANF/CNT-d. As seen in Figure S16, the CANF/CNT-d film has no obvious laminated structure, unlike the CANF/CNT aerogel film prepared by the aerogel approach. Because of the shrinkage during the drying process, the CANF/CNT-d film shows a thickness of 14 μm, which is smaller than the CANF/CNT aerogel films prepared by the ANF/CNT aerogel film. However, the CANF/CNT-d film shows a high density of 0.39 g/cm^3^ and a poor electrical conductivity of only 74 S/m (Figure S17), which is far smaller than the conductivity of the CANF/CNT-750 aerogel film (8540 S/m). The EMI SE of CANF/CNT-d is about 27.4 dB (Figure S18), which is also inferior to the EMI shielding performance of the CANF/CNT-750 aerogel film (Figure 3c). The above results show that the laminated densified structure can significantly enhance its electrical conductivity, resulting in excellent EMI shielding performance.

According to the EMI shielding theory, the EMI shielding performance is associated with the thickness of shielding materials. Therefore, the CANF/CNT-750 aerogel films with different thicknesses were prepared, and their EMI shielding performance was also studied, as shown in Figure 3d. Obviously, the EMI shielding performance of CANF/CNT-750 aerogel film gradually increases with the increase of film thickness. With the thickness of CANF/CNT-750 aerogel films increasing from 24 to 101 μm, the EMI SE increases from 36 to 54 dB. We also investigated the influence of CNTs contents on the EMI SE performance (Figure S19). When the content of CNTs increases from 10 to 20 wt %, the values of SE_T_ improve from 33.3 to 36 dB. However, continuing to increase the content of CNTs to 30 wt %, the EMI shielding performance has slightly enhanced, which may be caused by the threshold value of the conductive network formed by the CNTs (Figure S19b).

In order to meet the practical application, especially for the miniaturization of electronic devices, high EMI SE values are not the only factor for an EMI shielding material; the density of the material should also be considered. In terms of density, specific shielding effectiveness (SSE) was used to evaluate the EMI shielding performance for different materials.^65,66^ For CNT-based aerogel films, the SSE value can reach 481.5 dB cm^3^/g. However, the SSE cannot adequately estimate the EMI shielding performance. Because, as for a porous material, when its SSE is higher than other materials, it always needs a thicker work thickness. Therefore, SSE/*t* is more appropriate to judge the EMI shielding performance, which was determined via dividing SSE by the working thickness.^46,67^ Due to the densified fiber layer and porous structures, the CNT-based aerogel film shows an excellent EMI shielding performance and its values of SSE/*t* can reach 200 647.9 dB cm^2^/g, which outperforms other aerogel (Table S1) and CNT/polymer composites^68,69^ EMI shielding materials, demonstrating enormous potential as flexible and lightweight high-performance EMI shielding materials in practical applications.

The mechanisms of such CNT-based aerogel film can be illustrated in Figure 3f. The EMI shielding performance is mainly contributed to the excellent electrical conductivity of the densified laminated structure and porous structure. When the electromagnetic wave incident the surface of the CNT-based aerogel film, some electromagnetic waves are immediately reflected because of the free electrons on the surface of the CNT-based aerogel film. The rest of the electromagnetic waves transmit into the carbon film and interact with the electron, resulting in ohmic losses by currents. Due to the laminated structure, the electromagnetic waves passing through the first layer will repeat the attenuation behavior as the surface layer. This progress will repeat again and again until the electromagnetic wave is lost completely or transmitted through the film.

### Joule Heating Performance

**Figure 4:**
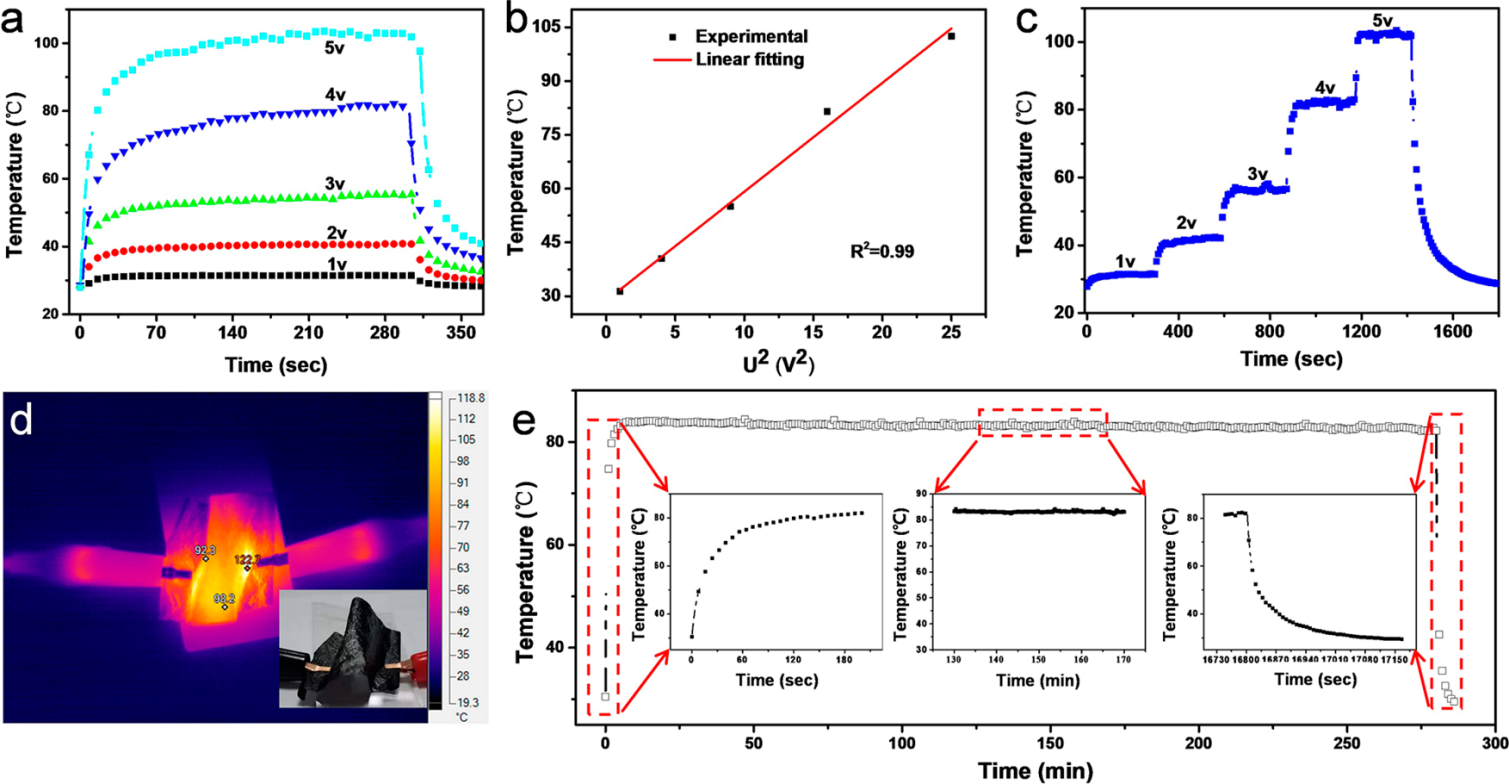
Joule heating performance of CNT-based aerogel films. (a) Time-dependent surface temperatures of the CANF/CNT-750 aerogel film under different input voltages. (b) Experimental and linear fitting of the relationship between surface temperatures and input voltages. (c) Surface temperatures of the CANF/CNT-750 aerogel film under step increased voltage. (d) Infrared images of the CANF/CNT-750 aerogel film under the bending condition and an input voltage of 5 V. (e) Long-term time-dependent heating performance of CANF/CNT-750 aerogel film under an input voltage of 4 V.

In addition to the high EMI shielding performance, the high electrical conductivity of such laminated porous CNT-based aerogel films also endows an outstanding Joule heating performance, quite applicable to the wearable heater. Figure 4a shows the surface temperatures of CANF/CNT-750 aerogel films under different applied voltages. With a low voltage of 2.0 V, the surface temperature increases from room temperature to 40.5 °C in 1 min, which is suitable for application in wearable thermal management devices, such as keeping warm or thermal therapy. For further increasing the input voltage, the surface temperature of CANF/CNT-750 aerogel film continues to increase. When the input voltage is increased to 5.0 V, the surface temperature of CANF/CNT-750 can reach 100.2 °C with a low input power density of 1.42 W/cm^2^. According to the Joule Law, expressed as the equation *Q* = *RI*^2^*t* (*Q* is the quantity of heat, *R* is the resistance, *I* is the current, and *t* is the heating time), the *Q* is proportional to the square of the current.^70,71^ As is well-known, *I* is proportional to the voltage under constant resistance. So, *Q* should be proportional to the voltage, if the resistance remains as a constant. From the *I*–*V* curves (Figure S20), the resistance of CANF/CNT-750 is stable with increased voltage. As shown in Figure 4b, the relationship between surface temperature and the square of input voltage was studied. The surface temperature of CANF/CNT-750 aerogel film presents an excellent linear relationship with the square of the input voltage, indicating a constant resistance of such a CNT-based aerogel film. Figure 4c shows the surface temperature of the CANF/CNT-750 aerogel film can be controlled by increasing the step voltage through the fast response of joule heating. Figure S21 shows the infrared images of the CANF/CNT-750 aerogel film under different input voltages. Clearly, a uniform temperature distribution can be seen from the thermal images, which is very important for wearable thermal management applications. Figure 4d shows the surface temperature of CANF/CNT-750 aerogel film under bending conditions. From the thermal image by the IR camera, the bent CANF/CNT-750 aerogel film also exhibits excellent Joule heating performance. Furthermore, the long-term time-dependent heating performance of CANF/CNT-750 aerogel film under an input voltage of 4 V was tested as shown in Figure 4e. It is noted that the CANF/CNT-750 aerogel film presents a stabilized surface temperature from 84.01 to 81.97 °C, indicating an excellent Joule heating stability.

## Conclusions

In summary, this work exploits a strategy to fabricate CNT-based aerogel films with laminated porous structures, which exhibit excellent electrical conductivity with low density and low thickness. Specifically, such laminated porous CNT-based aerogel films with densified fiber layers were fabricated from ANF and CNTs hybrid aerogel film through directional compression. Induced by stress, the three-dimensional network structure of ANFs and CNTs can be restructured into a laminated structure. Following carbonization treatment, the architecture with a higher porosity will be formed by the decomposition of ANFs. Attributing to the densified laminated structure and porous structure, such a CNT-based aerogel film exhibits a large specific surface area (341.9 m^2^/g) and high electrical conductivity (8540 S/m). The tensile strength of such CNT-based aerogel films is above 20 MPa, and its specific strength can be up to 117.6 MPa g^–1^ cm^3^. Owing to its high electrical conductivity, the CNT-based aerogel film offers an excellent EMI shielding performance of 35.9 dB, at a density and thickness of only 0.12 g/cm^3^ and 24 μm, respectively. Notably, the high specific shielding effectiveness is up to 200647.9 dB cm^2^/g, surpassing those of other reported aerogel electromagnetic interference shielding materials. The densified laminated structure ensures excellent electrical conductivity resulting in excellent EMI shielding performance. The porous laminated structure can effectively reduce density and induce multiple reflections of electromagnetic waves. The laminated CNT-based aerogel film with adjustable wetting properties also shows exceptional Joule heating performance profiting from the high electrical conductivity. We anticipate that such CNT-based aerogel films could be promising as lightweight EMI shielding materials and wearable electrical heaters. This work also gives an inspiration for the development of advanced aerogel materials for wearable applications.

## Methods

### Material

Aramid fiber (Kevlar 1000D) and carbon nanotubes (CNTs, purity >75 wt %, OCSiAl) were bought from Dupont company and ENUOEl Electronics Co., LTD, respectively. Dimethyl sulfoxide (DMSO), *tert*-butyl alcohol, and potassium *tert*-butoxide were purchased from Sinopharm Chemical Reagent Company. All the chemicals were used without further purification. DI water was collected from a Milli-Q water purification system.

### Preparation of Densified Carbon Aerogel Films

A certain amount of CNTs and potassium *tert*-butoxide were added into DMSO, followed by magnetic stirring and ultrasound until the carbon nanotubes were completely dispersed. Then, Kevlar was added into the CNTs solutions by magnetic stirring for 1 week, and the mass concentration of Kevlar is 1%. ANF/CNT (with the mass ratio of 10/1–10/3) solutions were treated into films through blade coating, and the films were immersed in DI water to form the hydrogel. The coating thickness varied from 500 to 1500 μm. The ANF/CNT hydrogel films were then treated by solvent exchange of a mix of tertiary butanol and DI water (1:1, volume). Following freeze-drying, ANF/CNT hybrid aerogel films were obtained. The as-prepared ANF/CNT hybrid aerogel films were compressed by press vulcanizer (ZRTB, ZB-910B) and then carbonized at different temperatures (550, 650, 750, 850, and 950 °C) under an argon atmosphere. The carbon aerogel films are denoted as CANF/CNT-550, CANF/CNT-650, CANF/CNT-750, CANF/CNT-850, and CANF/CNT-950. As a comparison, the ANF/CNT hydrogel film was directly dried by vacuum drying under 60 °C and carbonized under 750 °C, which was named CANF/CNT-d. The CANF/CNT films with different CNT concentrations were prepared with a similar procedure.

### Characterization on Morphology, Mechanical, and Electrical Properties

The morphology was investigated by scanning electron microscopy (SEM, Hitachi S-4800). The elements and structure were analyzed by X-ray diffraction (XRD, Bruker D8 Advance diffractometer, Cu Kα), small-angle X-ray scattering (SAXS, Nano STAR, Bruker-AXS), Raman spectrometer (LabRaM HR, Horiba Ltd.), and X-ray photoelectron spectroscopy (XPS, PHI 5000 VersaProbe II, ULVAC-PHI). The thermal decomposition behavior of aerogel film was studied by differential thermal analysis (TG 209F1 Libra thermogravimetric analyzer) and TG-FTIR (NETZSCH STA 449F5 STA449F5A-0235-M) under an argon atmosphere. The contact angles were tested by an optical contact angle goniometer (OCA 15EC, Data Physics Instruments). The mechanical properties were carried out with an Instron universal test instrument (Model 5576, Instron Corporation). The specific surface area and the pore size distribution of the samples were tested by the Brunauer–Emmett–Teller (BET) method and Barrett–Joyner–Halenda (BJH) method (ASAP 2020, Micromeritics). The densities of carbon aerogel films were calculated from mass and volume, by cutting the film into 1 cm × 1 cm. The electrical conductivity and electrical behavior were carried out by a four-probe resistivity meter (Model ST2258C, Jingge). The voltage was input by Precision Measurement DC Supply (2280S-60-3, Keithley), and the temperature was collected by Data Acquisition/Switch Unit (34970A, Keysight).

### Characterization of EMI Shielding Performances

The transmission line method was used to evaluate the EMI shielding performance of carbon aerogel films. S-parameter was collected by a vector network analyzer (N5227A) in the frequency range of 8.2–12.4 GHz (X-band) through a waveguide method. The shielding effectiveness was calculated on the basis of the s-parameter output by a vector network analyzer.

## Supporting Information Available

Discussions of EMI shielding measurements, orientation measurements, and input power density measurements, figures of WAXS patterns and its relative radial profiles, SEM images, stress–strain curves, surface morphology, FT-IR spectra, elemental content, Raman spectra, XRD patterns, specific surface area, pore size distribution curves, contact angle images, comparison of experimentally and theoretically results for CANF/CNT-750, density and electric conductivity, electrical conductivity *I*–*V* curves, and IR camera images, and tables of EMI shielding performance and specific EMI shielding performance (PDF)
