# Supplementary material for: Laminated Structural Engineering Strategy toward Carbon Nanotube-Based Aerogel Films
Source: ACS Nano. 2022 May 19;16(6):9378–88. doi: 10.1021/acsnano.2c02193 (PMC9245345; doi:10.1021/acsnano.2c02193)
Supplement: Supplementary file 1 — nn2c02193_si_001.pdf [file nn2c02193_si_001.pdf]

Supporting Information

Laminated Structural Engineering Strategy toward Carbon Nanotube-Based Aerogel Films

*Chen Fu[a], Zhizhi Sheng[a], and Xuetong Zhang[a, b]**

[a]Suzhou Institute of Nano-tech and Nano-bionics, Chinese Academy of Sciences, Suzhou 215123, P. R. China

[b]Division of Surgery & Interventional Science, University College London, London NW3 2PF, UK

*E-mail: xtzhang2013@sinano.ac.cn or xuetong.zhang@ucl.ac.uk

1. Electromagnetic Interference (EMI) Shielding Measurements

Electromagnetic interference shielding measurements of CNT-based aerogel films were carried out in a WR-90 rectangular waveguide using a 2-port network analyzer (N5227A) in the X-band frequency range (8.2-12.4 GHz). The samples were cut into a rectangular shape in a dimension of 24 × 12 mm^2, which is slightly larger than the sample holder dimension (22.84 × 10.14 mm^2). Electromagnetic interference shielding efficiency can be calculated according to the following formula:[1]

$$R = |s_{11}|^2 = |s_{22}|^2 \quad (1)$$

$$T = |s_{12}|^2 = |s_{21}|^2 \quad (2)$$

$$SE_R = 10\log\left(\frac{1}{1-R}\right) = 10\log\left(\frac{1}{1-|s_{11}|^2}\right) \quad (3)$$

$$SE_A = 10\log\left(\frac{1-R}{T}\right) = 10\log\left(\frac{1-|S_{11}|^2}{|S_{21}|^2}\right) \quad (4)$$

$$SE_T = SE_R + SE_A + SE_{MR} \quad (5)$$

where R, A, T are the reflection coefficient, absorption coefficient, and transmission coefficient, respectively; S_{ij} represents the s-parameter obtained by the network analyzer; SE_R, SE_A, SE_T, and SE_{MR} are the reflection effectiveness, absorption effectiveness, total shielding effectiveness, and multiple internal reflection effectiveness, respectively.

Specific shielding effectiveness (SSE) is derived to compare the effectiveness of shielding materials by taking into account the density, which can be obtained by dividing the EMI SE by the density of materials as follows:[1]

$$\text{SSE=EMI SE/density =dB cm}^3\text{ g}^{-1}. \quad (6)$$

However, SSE has a basic limitation, that is, it does not account for the thickness information. So, the absolute effectiveness (SSE/t) was obtained, which is take into account the thickness:[1]

$$SSE_t = SSE/t = \text{dB cm}^2\text{ g}^{-1}. \quad (7)$$

2. Orientation measurements for aerogel film

Herman's orientation factor (f) was calculated to describe the degree of orientation of the nanofibers as well as CNTs relative to the film plane using Equation (3):[2]

$$f = \frac{3\langle cos^2\emptyset\rangle - 1}{2} \quad (8)$$

where the mean-square cosine is calculated from the scattered intensity I(ø) by integrating over the azimuthal angle ø according to Equation (4):[2]

$$\langle cos^2\emptyset\rangle = \frac{\int_0^{\frac{\pi}{2}} I(\emptyset)sin\emptyset cos^2\emptyset d\emptyset}{\int_0^{\frac{\pi}{2}} I(\emptyset)sin\emptyset d\emptyset} \quad (9)$$

where ø is the angle between the film plane and the nanofibers as well as CNTs. f = 1 when all nanofibers and CNTs are parallel to the film plane and f = 0 for random orientation of

nanofibers and CNTs.

3. Input power density for electrothermal conversion

The voltage was input by Precision Measurement DC Supply and the corresponding current could be obtained simultaneously. The CNT-based aerogel film was cut into a certain size (1×2 cm^2). Assuming that electrical energy is completely converted into heat, the input power density (P_A) can be calculated by Joule's law with the following formula:[2]

$$P_A = \frac{UI}{A} \tag{10}$$

Where U is the input voltage, I is the current through the aerogel film and A is the area of the aerogel film.

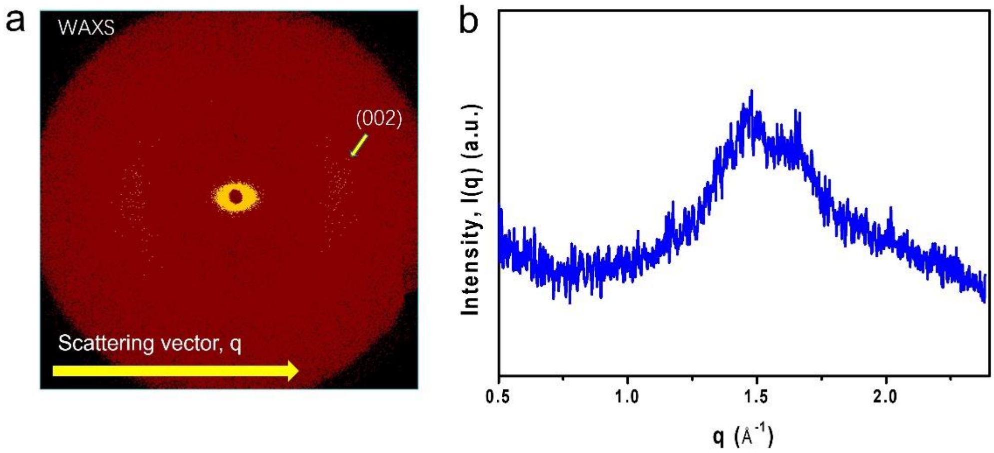

Figure S1. Typical WAXS patterns (a) and its relative radial profiles (b) of D-ANF/CNT film.

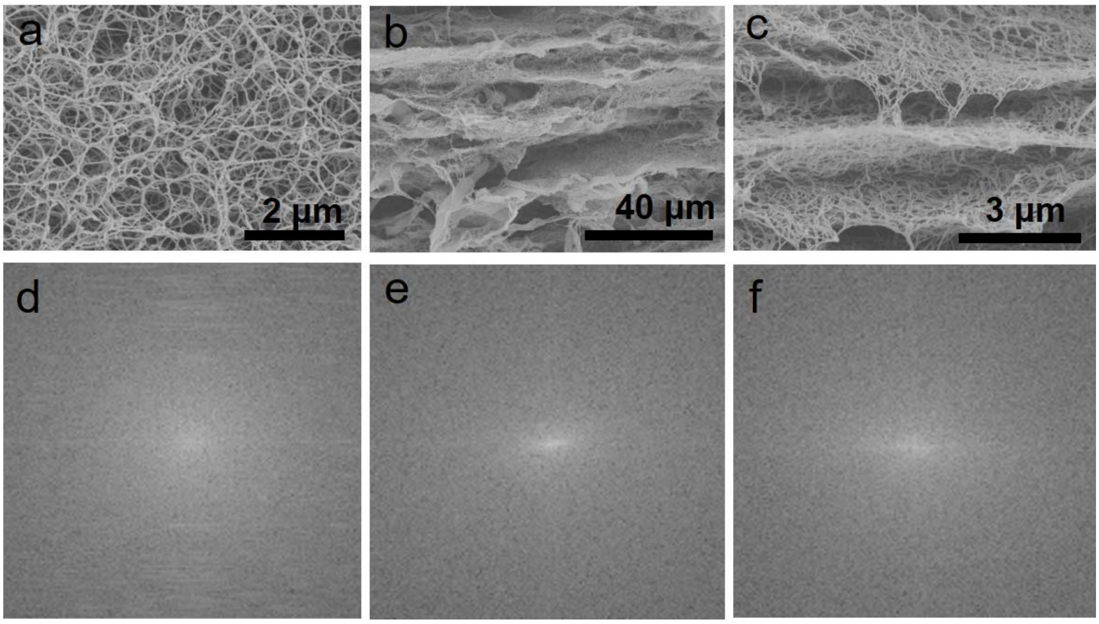

Figure S2. SEM images of ANF/CNT aerogel films with different compressive treatments and their corresponding fast Fourier transform (FFT) frequency domain images: 0 MPa (a and d), 10 MPa (b and e), and 20 MPa (c and f).

The fast Fourier transform (FFT) method was used to analyze the structural evolution of ANF/CNT aerogel films with different compressive strain treatments. Orientation Factor was calculated from the standard formulation as defined by $F = \langle cos^2\emptyset \rangle = \frac{\int_0^{\frac{\pi}{2}} I(\emptyset) cos^2\emptyset d\emptyset}{\int_0^{\frac{\pi}{2}} I(\emptyset) d\emptyset}$, where I is the intensity profile as a function of the angle between the structural unit vector and the reference direction.[3] In this case, the intensity profile was derived from the FFT of side-view SEM images. Without compression, the nanofibers are randomly oriented and there is a circular spot on the FFT frequency domain image (Figure S2d). After compressive treatment under 10 MPa and 20 MPa, the randomly aligned nanofibers deformed into the orientated structure (Figure S2b and S2c). From the FFT frequency domain images, the plot also has a prominent orientation (Figure S2e and S2f).

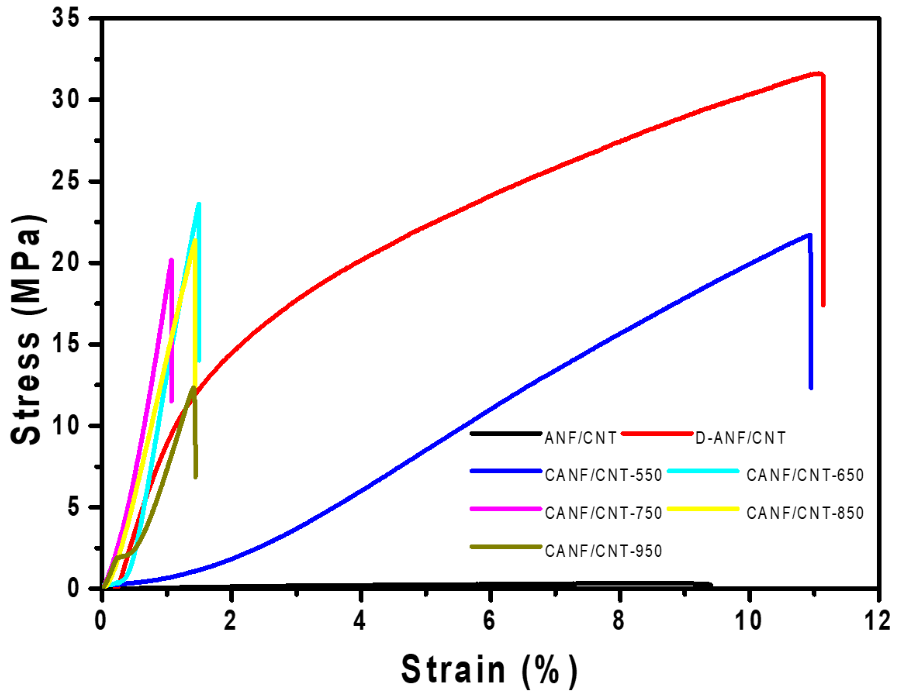

Figure S3. Tensile stress-strain curves of ANF/CNT aerogel film, D-ANF/CNT film and CANF/CNT aerogel films at different temperatures.

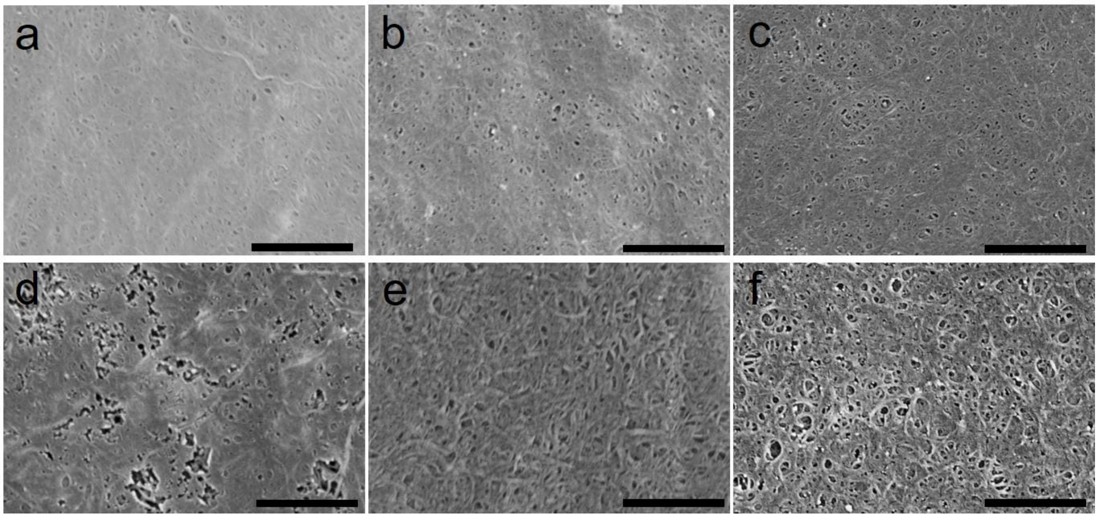

Figure S4. The surface morpholgoy of ANF/CNT (a) and CANF/CNT with different carbonization temperatures: 550 ºC(b), 650 ºC (c), 750 ºC (d), 850 ºC (e), and 950 ºC (f), (scale bar: 1 μm).

Without carbonization, the ANF/CNT aerogel film exhibit a smooth surface structure with

some small pores (Figure S4a). Following the carbonized temperature increase, the pores on the CANF/CNT films got bigger and bigger, due to the pyrolysis of ANF and the generated gases. For CANF/CNT-550 (Figure S4b) and CANF/CNT-650 (Figure S4c), a continuous smooth surface can be observed. When the carbonization temperature increases to 750 ºC, a more porous structure can be observed on the surface (Figure S4d). For CANF/CNT-850 (Figure S4e) and CANF/CNT-950 (Figure S4f), the smooth surface structures were destroyed and obvious nanofibers network structure can be observed.

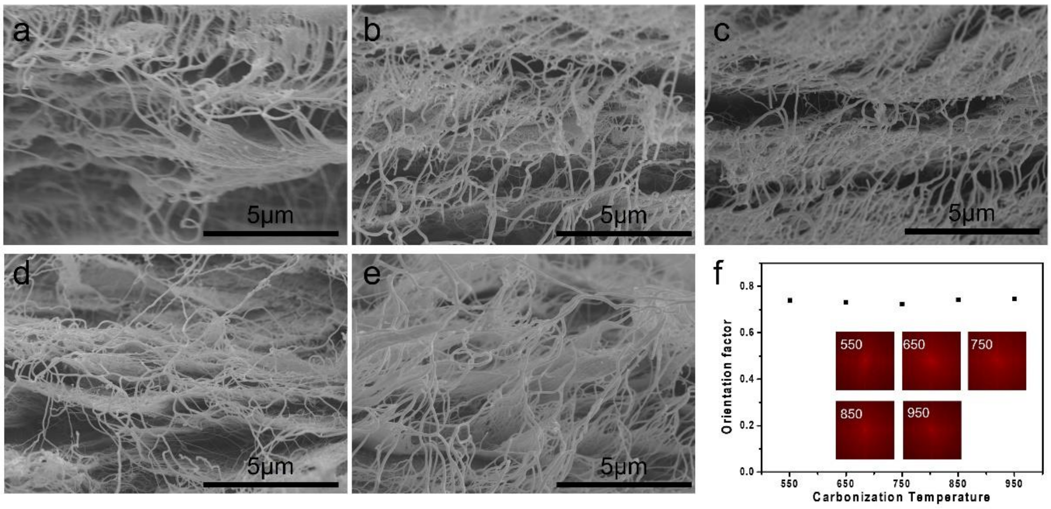

Figure S5. The SEM images of CANF/CNT-550 (a), CANF/CNT-650 (b), CANF/CNT-750 (c), CANF/CNT-850 (d), and CANF/CNT-950 (e), and their Orientation factors (f, insets: 2D FFT of the SEM images).

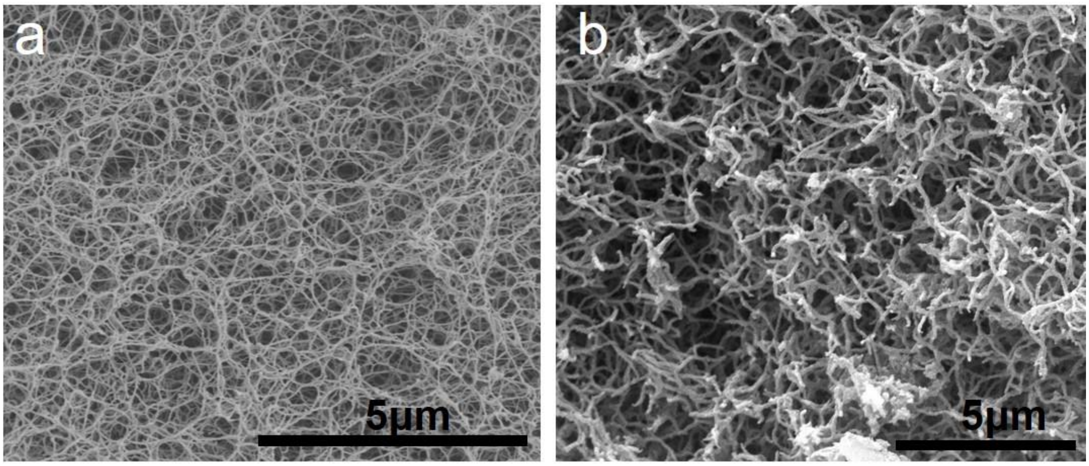

Figure S6. The SEM images of pure ANF aerogel film before (a) and after carbonization at 950 ºC (b).

In order to investigate the morphology of ANF after carbonization, we prepared a pure ANF aerogel film without CNTs and the ANF aerogel film was carbonized under 950 ºC by the protection of Ar. From the SEM image, the pure ANF aerogel film shows a three-dimensional network structure of nanofibers (Figure S6a), and the nanofiber morphology of ANF was maintained after carbonization treatment (Figure S6b).

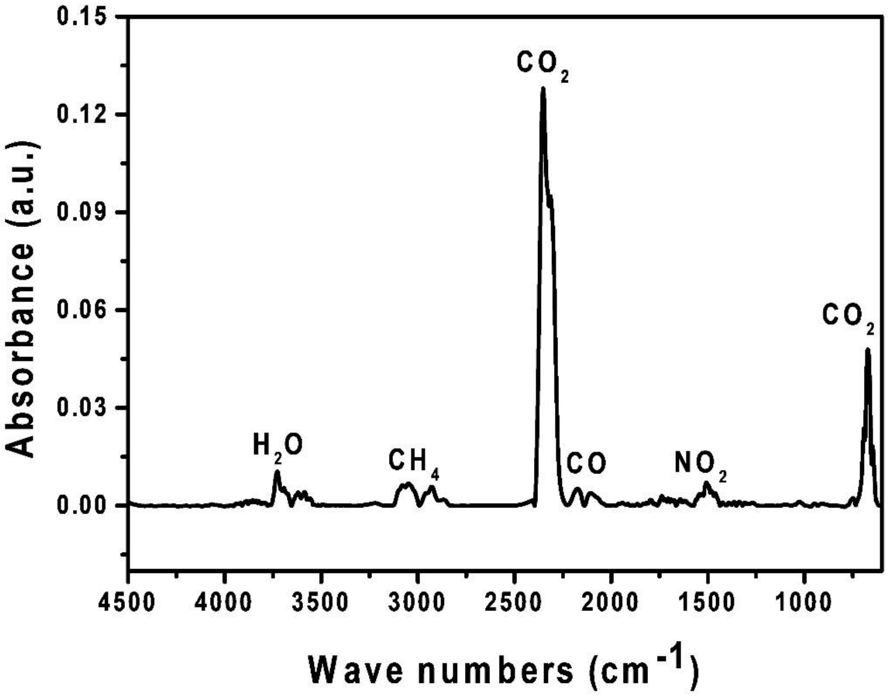

Figure S7. The FTIR spectrum of decomposed gases at 638 ºC.

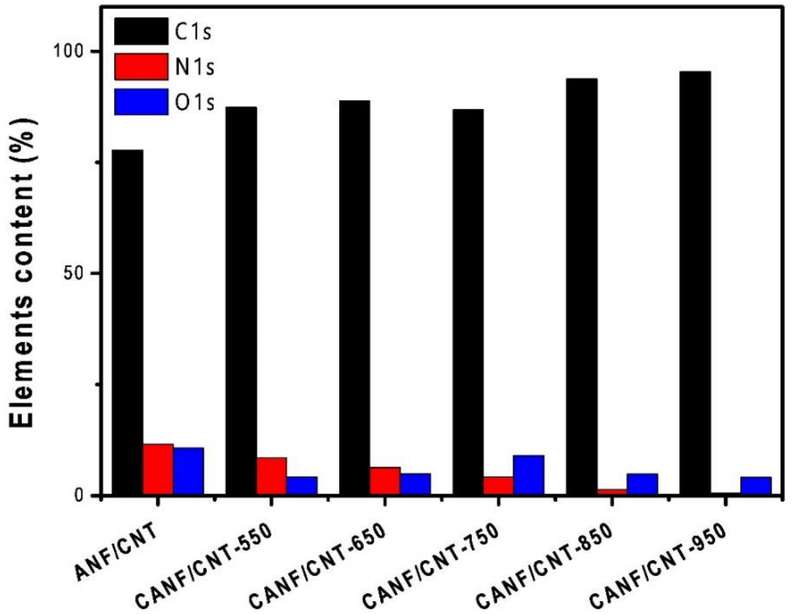

Figure S8. The elemental content of C, N, and O for ANF/CNT, CANF/CNT-550, CANF/CNT-650, CANF/CNT-750, CANF/CNT-850, and CANF/CNT-950, respectively.

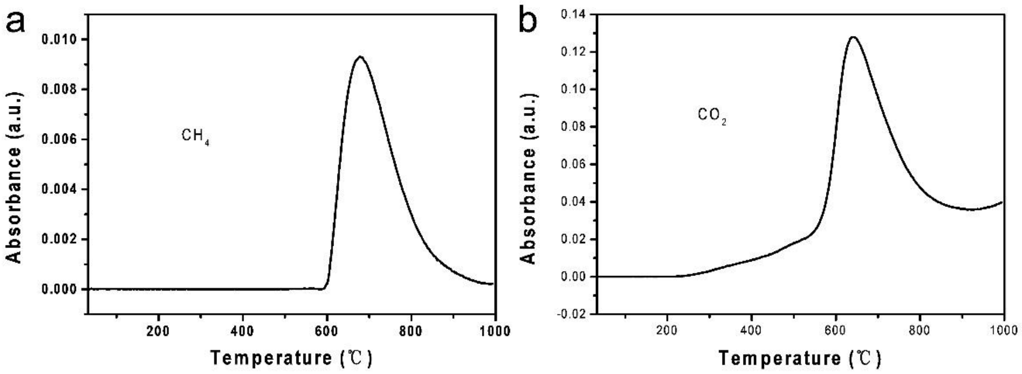

Figure S9. The FTIR spectrum of CH_4 and CO_2 is generated during the pyrolysis of ANF/CNT.

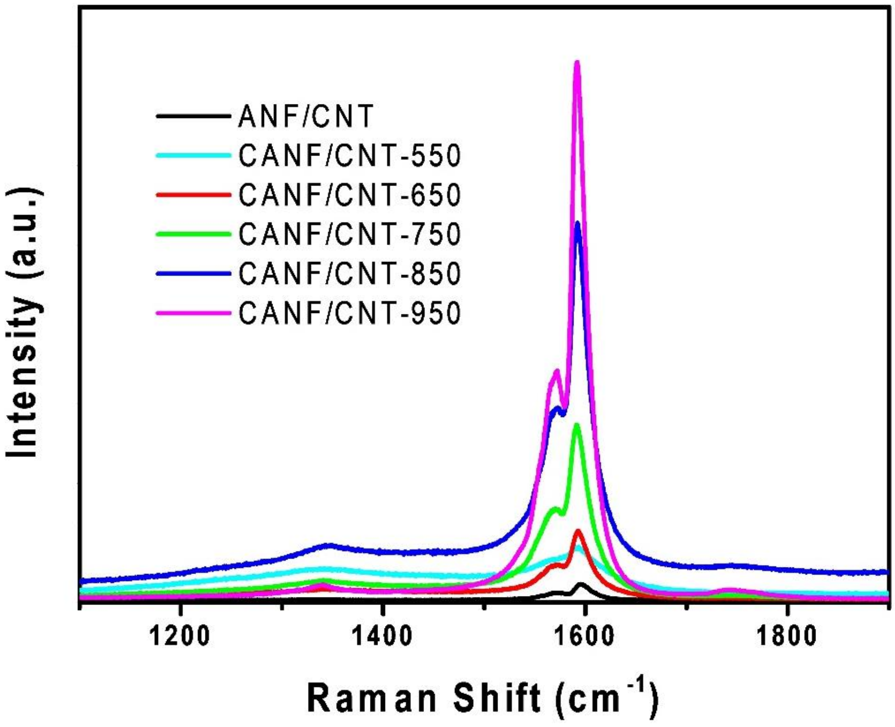

Figure S10. The Raman spectrum of CANF/CNT with different carbonization temperatures.

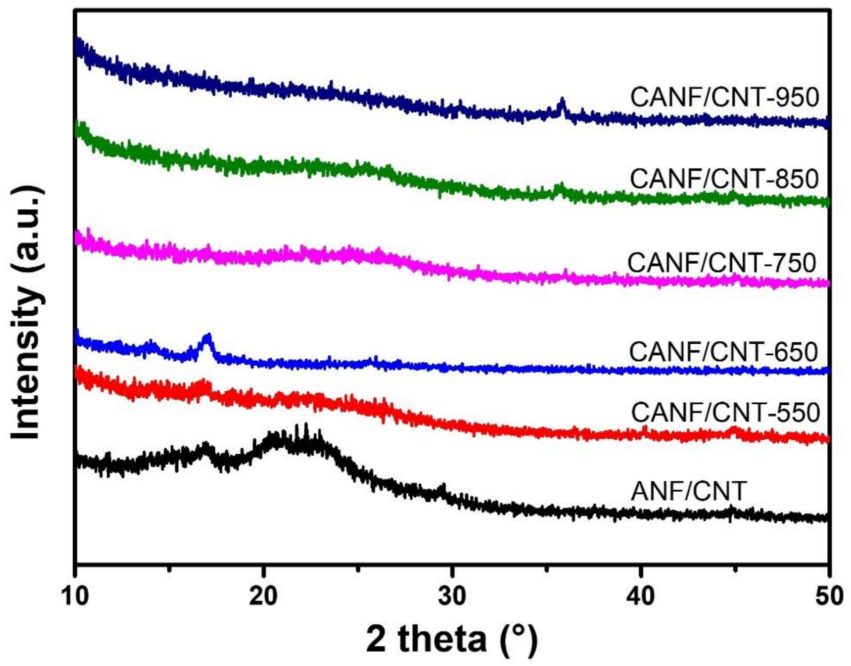

Figure S11. The XRD spectra of CANF/CNT with different carbonization temperatures.

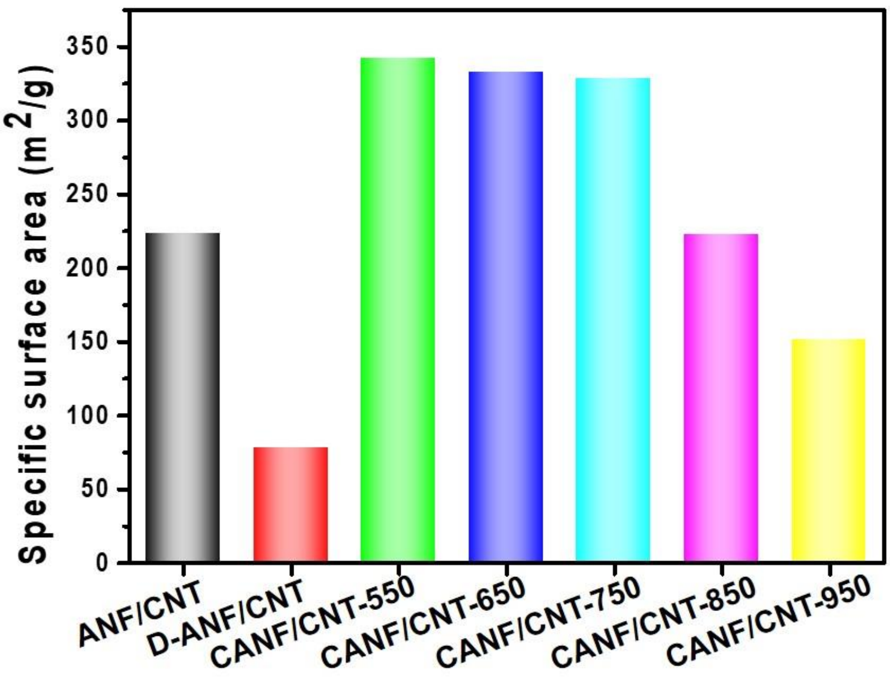

Figure S12. The specific surface area of ANF/CNT, D-ANF/CNT, and CANF/CNT with different carbonization temperatures.

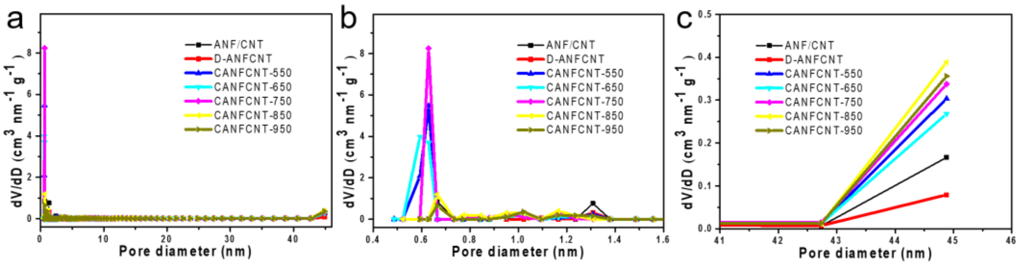

Figure S13. The pore size distribution curves (a), micropore distribution (b), and mesoporous (c) of CNT-based aerogel films carbonized at different temperatures.

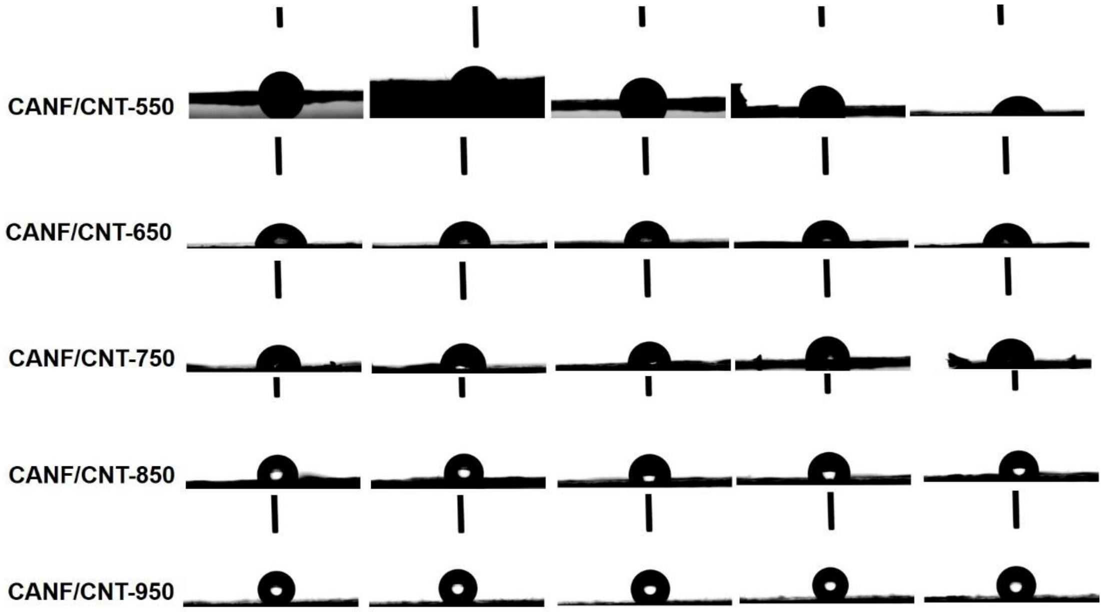

Figure S14. The contact angle of CANF/CNT aerogel films with different carbonization temperatures.

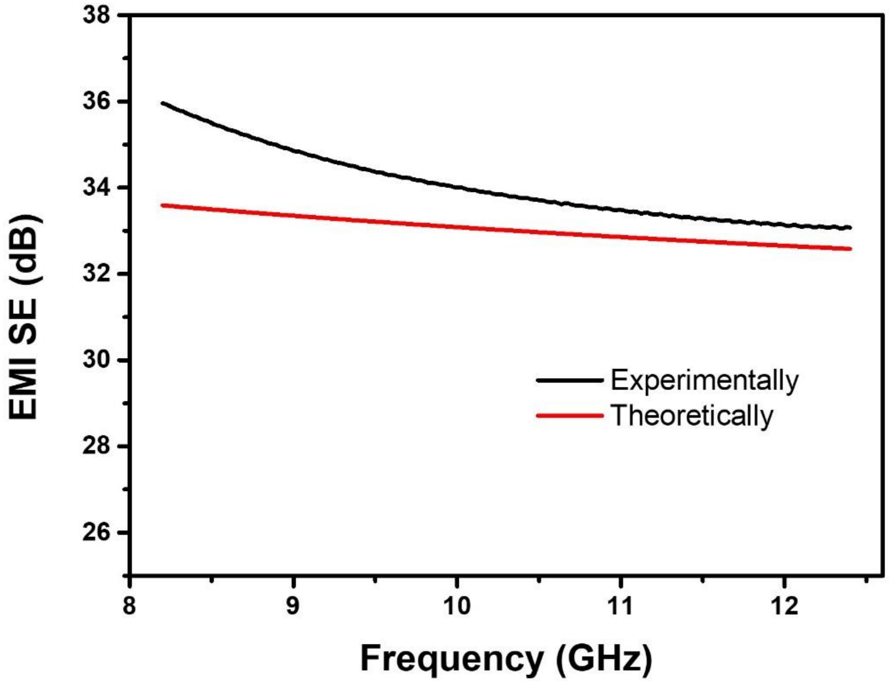

Figure S15. The comparison of experimentally and theoretically results for CANF/CNT-750.

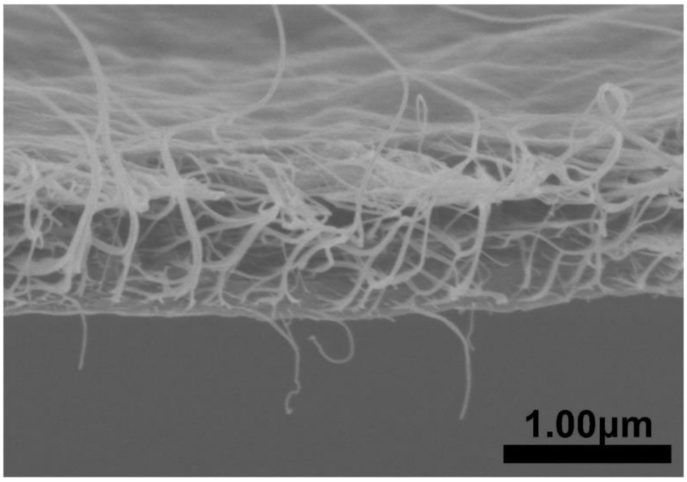

Figure S16. the cross-section SEM image of CANF/CNT-d.

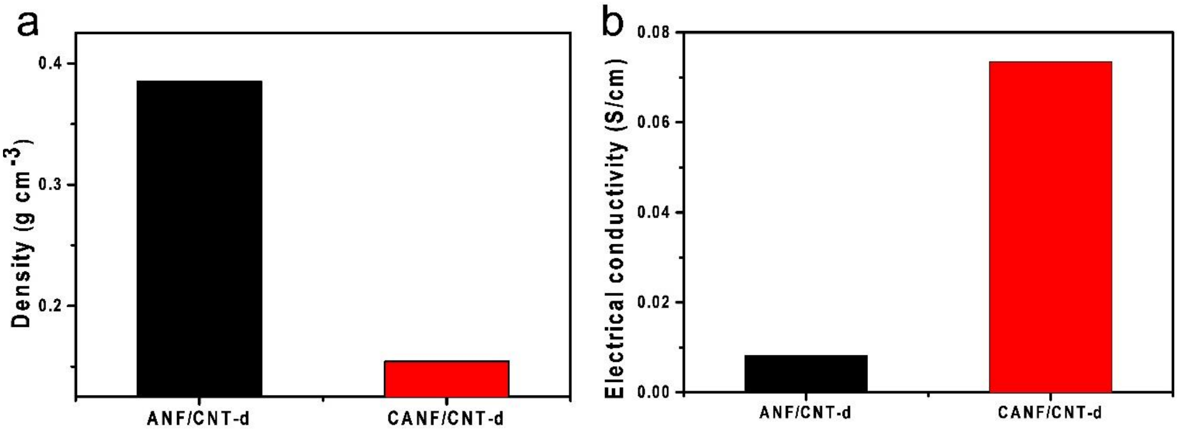

Figure S17. The density (a) and electric conductivity (b) of the carbon film without aerogel progress.

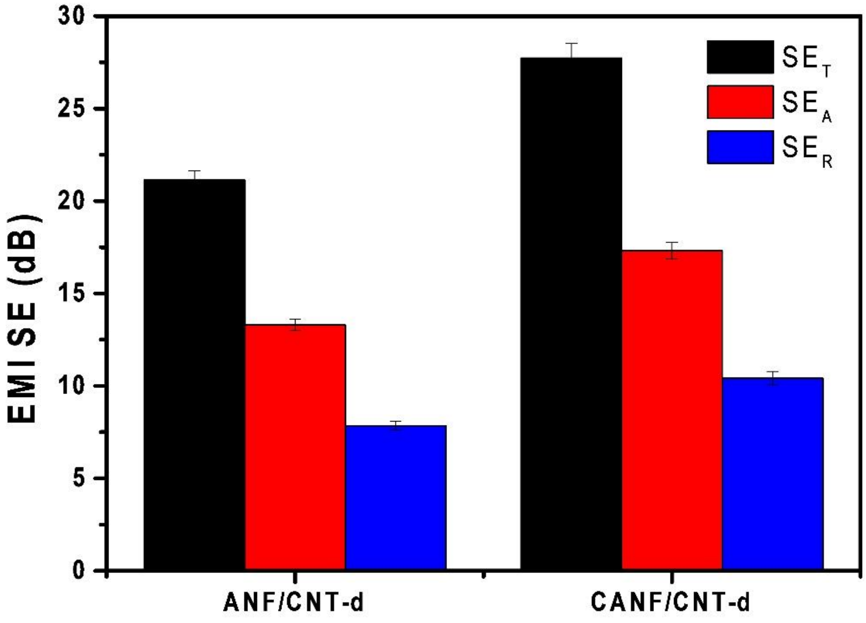

Figure S18. The EMI SE of the CNT-based film without aerogel progress before (RT) and after carbonization progress (750℃).

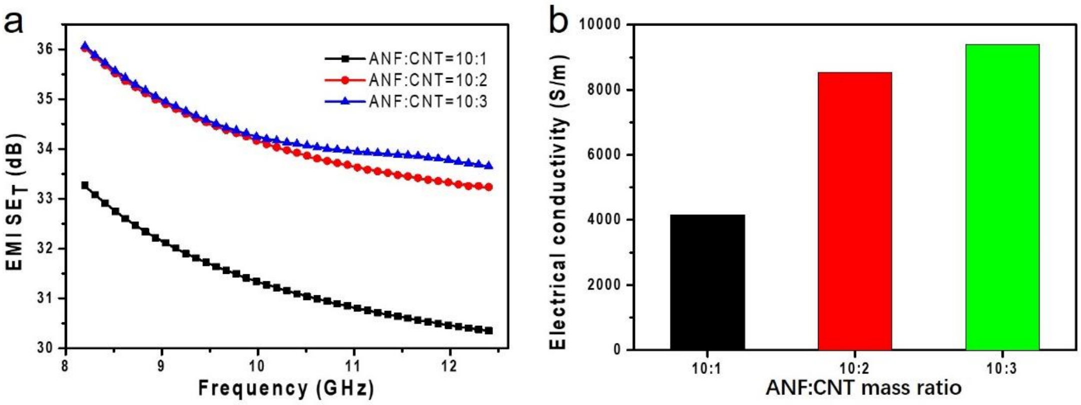

Figure S19. The EMI SE$_T$ (a) and electrical conductivity (b) of CANF/CNT aerogel films with different ANF:CNT mass ratios in the processing recipe.

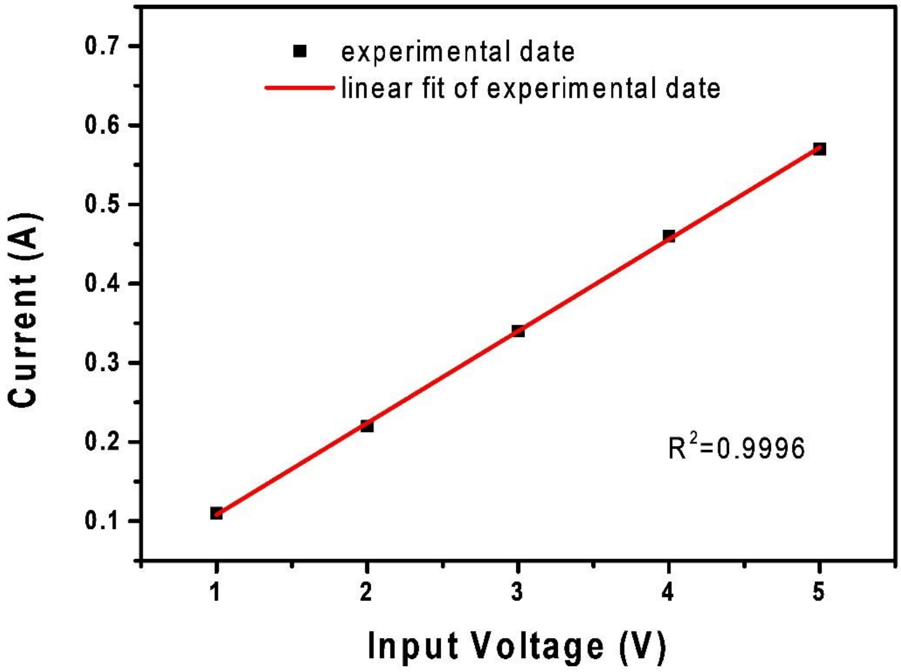

Figure S20. The I-V curves for CANF/CNT-750 aerogel film.

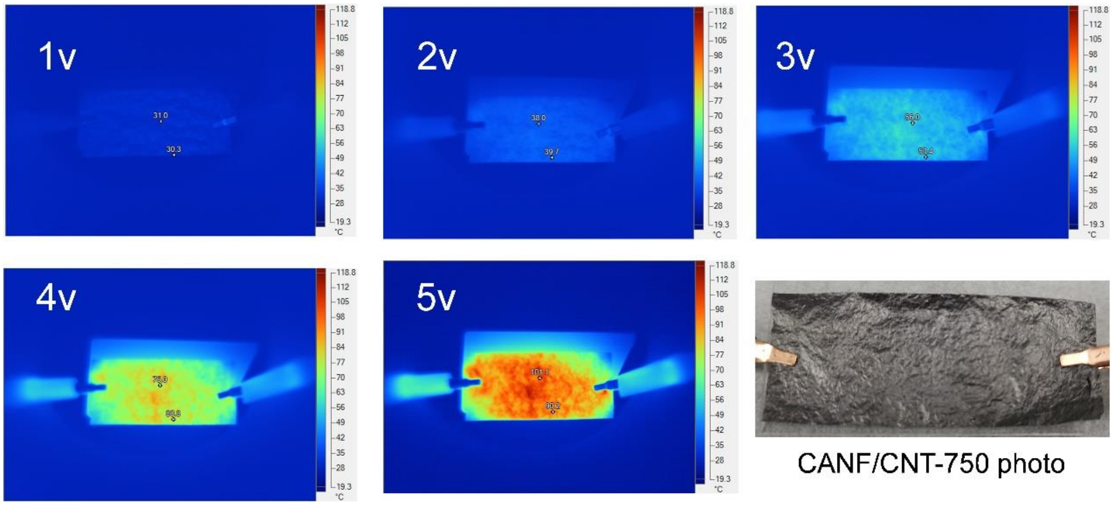

Figure S21. The IR camera images of CANF/CNT-750 aerogel film under different input voltages and its photo.

Table S1. EMI SE of various aerogel shielding materials.

	Sample	Density ($g \cdot cm^{-3}$)	Thickness (mm)	Electrical conductivity ($S \cdot m^{-1}$)	EMI SE_T (dB)	SSE/t ($dB \cdot cm^2 \cdot g^{-1}$)	Ref.
Graphene	Graphene/polyimide	0.076	2.5	2.58	28.8	1515.8	4
	Graphene/carbon	0.002	2	1000	21.3	53250.0	5
	Graphene/texture	0.07	2	8000	27	1928.6	6
	Graphene/cellulose	0.00283	5	2.57	47.8	33780.9	7
	Graphene	0.41	0.12	8000	105	21341.5	8
	Graphene	0.006	5	78.3	40.5	13500.0	9
	Cu Nanowire@Graphene	0.0165	9.46	2083	52.5	3363.4	10
	Cellulose nanofibril/RGO	0.0058	3	64.49	33	18965.5	11
	Graphene	0.122	1.547		53	2808.2	12
	Graphene/silver nanowires	0.019	5		45.2	4757.9	13
Carbon	Carbon	0.079	2	44.3	93.1	5892.4	14
	CNF@Co/C	0.023	4	0.47	56.07	6094.6	15
	Carbon	0.0544	0.162	1029.5	41.4	46977.1	16
	Carbon	0.00125	4		36.75	73500.0	17
	Carbon	0.057	4	1.35	40	1754.4	18
Mxene	MXene@wood	0.108	10	37.04	71.8	664.8	19
	PVA/MXene	0.034	3.49	314	53.3	4491.8	20
	MXene/CNT	0.0091	2	447.2	51	28022.0	21

	WPU/MXene/NiFe2O4	0.0073	2	226.4	22.8	15620.0	22
	Cellulose nanofibril/Mxene	0.0221	2		62.91	14230.0	23
	MXene/rGO	0.032	2	1085	56.4	8812.5	24
	MXene/CNT	0.042	3	943	103.9	8246.0	25
	Nanocellulose-MXene	0.0015	1	27.6	28.41	189400.0	26
	MXene/Carbon	0.2483	3.23	28.34	70	872.8	27
	MXene	0.0055	1		48.5	88181.8	28
	MXene/PEDOT:PSS	0.012	4.5	3.097	59	10841.0	29
CNT	Nanocellulose/CNT	0.072	2	26.2	39.8	2763.9	30
	Aramid Nanofiber/CNT	0.043	0.568	230	54.4	22273.2	31
	MWCNT/Cellulose	0.095	2.5	1.8	20.8	875.8	32
Others	V_2O_5/polyaniline	0.02	6	1.69	34	2833.3	33
	Nanocellulose/Silver Nanowire	0.0017	1	416	30.3	178235.3	34
CNT	CANF/CNT-550	0.17	0.024	1408.5	25.97	63652.0	This work
	CANF/CNT-650	0.15	0.024	3449.4	31.33	87027.8	
	CANF/CNT-750	0.12	0.024	8540.5	35.95	124826.4	
	CANF/CNT-850	0.087	0.024	6561.6	34.72	166283.5	
	CANF/CNT-950	0.058	0.024	2490.9	27.87	200215.5	

REFERENCES

(1) Shahzad, F.; Alhabeb, M.; Hatter, C.; Anasori, B.; Hong, S. M.; Koo, C. M.; Gogotsi, Y.; Electromagnetic Interference Shielding with 2D Transition Metal Carbides (MXenes). *Science* **2016**, *353*, 1137-1140.

(2) Li, S.; Fan, Z.; Wu, G.; Shao, Y.; Xia, Z.; Wei, C.; Shen, F.; Tong, X.; Yu, J.; Chen, K.; Wang, M.; Zhao, Y.; Luo, Z.; Jian, M.; Sun, J.; Kaner, R. B.; Shao, Y.; Assembly of Nanofluidic MXene Fibers with Enhanced Ionic Transport and Capacitive Charge Storage by Flake Orientation. *ACS Nano* **2021**, *15*, 7821-7832.

(3) Dai, Z.; Liu, L.; Qi, X.; Kuang, J.; Wei, Y.; Zhu, H.; Zhang, Z.; Three-Dimensional

Sponges with Super Mechanical Stability: Harnessing True Elasticity of Individual Carbon Nanotubes in Macroscopic Architectures. *Sci. Rep.* **2016**, *6*, 18930.

(4) Yu, Z.; Dai, T.; Yuan, S.; Zou, H.; Liu, P.; Electromagnetic Interference Shielding Performance of Anisotropic Polyimide/Graphene Composite Aerogels. *ACS Appl. Mater. Interfaces* **2020**, *12*, 30990-31001.

(5) Zeng, Z.; Wang, C.; Zhang, Y.; Wang, P.; Seyed Shahabadi, S. I.; Pei, Y.; Chen, M.; Lu, X.; Ultralight and Highly Elastic Graphene/Lignin-Derived Carbon Nanocomposite Aerogels with Ultrahigh Electromagnetic Interference Shielding Performance. *ACS Appl. Mater. Interfaces* **2018**, *10*, 8205-8213.

(6) Song, W; Guan, X; Fan, L; Cao, W; Wang, C; Cao, M; Tuning Three-Dimensional Textures with Graphene Aerogels for Ultra-Light Flexible Graphene/Texture Composites of Effective Electromagnetic Shielding. *Carbon* **2015**, *93*, 151-160.

(7) Wan, Y; Zhu, P; Yu, S; Sun, R.; Wong, C; Liao, W; Ultralight, Super-Elastic and Volume-Preserving Cellulose Fiber/Graphene Aerogel for High-Performance Electromagnetic Interference Shielding. *Carbon* **2017**, *115*, 629-639.

(8) Xi, J.; Li, Y.; Zhou, E.; Liu, Y.; Gao, W.; Guo, Y.; Ying, J.; Chen, Z.; Chen, G.; Gao, C.; Graphene Aerogel Films with Expansion Enhancement Effect of High-Performance Electromagnetic Interference Shielding. *Carbon* **2018**, *135*, 44-51.

(9) González, M.; Baselga, J.; Pozuelo, J.; Modulating the Electromagnetic Shielding Mechanisms by Thermal Treatment of High Porosity Graphene Aerogels. *Carbon* **2019**,

147, 27-34.

(10) Wu, S.; Zou, M.; Li, Z.; Chen, D.; Zhang, H.; Yuan, Y.; Pei, Y.; Cao, A.; Robust and Stable Cu Nanowire@Graphene Core-Shell Aerogels for Ultraeffective Electromagnetic Interference Shielding. *Small* **2018**, *14*, 1800634.

(11) Li, M.; Han, F.; Jiang, S.; Zhang, M.; Xu, Q.; Zhu, J.; Ge, A.; Liu, L.; Lightweight Cellulose Nanofibril/Reduced Graphene Oxide Aerogels with Unidirectional Pores for Efficient Electromagnetic Interference Shielding. *Adv. Mater. Interfaces* **2021**, *8*, 2101437.

(12) Han, D.; Zhao, Y; Bai, S; Ping W. C.; High Shielding Effectiveness of Multilayer Graphene Oxide Aerogel Film/Polymer Composites. *RSC Adv.* **2016**, *6*, 92168-92174.

(13) Liu, X.; Chen, T.; Liang, H.; Qin, F.; Yang, H.; Guo, X.; Facile Approach for a Robust Graphene/Silver Nanowires Aerogel with High-Performance Electromagnetic Interference Shielding. *RSC Adv.* **2019**, *9*, 27-33.

(14) Zhou, Z; Liang, Y.; Huang, H; Li, L.; Yang, B.; Li, M; Yan, D; Lei, J.; Li, Z; Structuring Dense Three-Dimensional Sheet-Like Skeleton Networks in Biomass-Derived Carbon Aerogels for Efficient Electromagnetic Interference Shielding. *Carbon* **2019**, *152*, 316-324.

(15) Fei, Y.; Liang, M.; Zhou, T.; Chen, Y.; Zou, H.; Unique Carbon Nanofiber@ Co/C Aerogel Derived Bacterial Cellulose Embedded Zeolitic Imidazolate Frameworks for High-Performance Electromagnetic Interference Shielding. *Carbon* **2020**, *167*, 575-584.

(16) Zhou, B.; Han, G.; Zhang, Z.; Li, Z.; Feng, Y.; Ma, J.; Liu, C.; Shen, C.; Aramid Nanofiber-Derived Carbon Aerogel Film with Skin-Core Structure for High Electromagnetic Interference Shielding and Solar-Thermal Conversion. *Carbon* **2021**, *184*, 562-570.

(17) Liao, D.; Guan, Y.; He, Y.; Li, S.; Wang, Y.; Liu, H.; Zhou, L.; Wei, C.; Yu, C.; Chen, Y.; Pickering Emulsion Strategy for High Compressive Carbon Aerogel as Lightweight Electromagnetic Interference Shielding Material and Flexible Pressure Sensor. *Ceram. Int.* **2021**, *47*, 23433-23443.

(18) Vazhayal, L.; Wilson, P.; Prabhakaran, K.; Waste to Wealth: Lightweight, Mechanically Strong and Conductive Carbon Aerogels from Waste Tissue Paper for Electromagnetic Shielding and CO_2 Adsorption. *Chem. Eng. J.* **2020**, *381*, 122628.

(19) Zhu, M.; Yan, X.; Xu, H.; Xu, Y.; Kong, L.; Ultralight, Compressible, and Anisotropic MXene@Wood Nanocomposite Aerogel with Excellent Electromagnetic Wave Shielding and Absorbing Properties at Different Directions. *Carbon* **2021**, *182*, 806-814.

(20) Guo, Z.; Li, Y.; Jin, P.; Zhang, T.; Zhao, Y.; Ai, Y.; Xiu, H.; Zhang, Q.; Fu, Q.; Poly(vinyl alcohol)/MXene Biomimetic Aerogels with Tunable Mechanical Properties and Electromagnetic Interference Shielding Performance Controlled by Pore Structure. *Polymer* **2021**, *230*, 124101.

(21) Deng, Z.; Tang, P.; Wu, X.; Zhang, H; Yu, Z.; Superelastic, Ultralight, and Conductive $Ti_3C_2T_x$ MXene/Acidified Carbon Nanotube Anisotropic Aerogels for Electromagnetic

Interference Shielding. *ACS Appl. Mater. Interfaces* **2021**, *13*, 20539-20547.

(22) Wang, Y.; Qi, Q.; Yin, G.; Wang, W.; Yu, D.; Flexible, Ultralight, and Mechanically Robust Waterborne Polyurethane/$Ti_3C_2T_x$ MXene/Nickel Ferrite Hybrid Aerogels for High-Performance Electromagnetic Interference Shielding. *ACS Appl. Mater. Interfaces* **2021**, *13*, 21831-21843.

(23) Li, Y.; Chen, Y.; He, X.; Xiang, Z.; Heinze, T.; Qi, H.; Lignocellulose Nanofibril/Gelatin/MXene Composite Aerogel with Fire-Warning Properties for Enhanced Electromagnetic Interference Shielding Performance. *Chem. Eng. J.* **2022**, *431*, 133907.

(24) Zhao, S.; Zhang, H. B.; Luo, J. Q.; Wang, Q. W.; Xu, B.; Hong, S.; Yu, Z. Z.; Highly Electrically Conductive Three-Dimensional $Ti_3C_2T_x$ MXene/Reduced Graphene Oxide Hybrid Aerogels with Excellent Electromagnetic Interference Shielding Performances. *ACS Nano* **2018**, *12*, 11193-11202.

(25) Sambyal, P.; Iqbal, A.; Hong, J.; Kim, H.; Kim, M. K.; Hong, S. M.; Han, M.; Gogotsi, Y.; Koo, C. M.; Ultralight and Mechanically Robust $Ti_3C_2T_x$ Hybrid Aerogel Reinforced by Carbon Nanotubes for Electromagnetic Interference Shielding. *ACS Appl. Mater. Interfaces* **2019**, *11*, 38046-38054.

(26) Zeng, Z.; Wang, C.; Siqueira, G.; Han, D.; Huch, A.; Abdolhosseinzadeh, S.; Heier, J.; Nuesch, F.; Zhang, C. J.; Nystrom, G.; Nanocellulose-MXene Biomimetic Aerogels with Orientation-Tunable Electromagnetic Interference Shielding Performance. *Adv. Sci.*

2020, *7*, 2000979.

(27) Li, S.; Wang, J.; Zhu, Z.; Liu, D.; Li, W.; Sui, G.; Park, C. B.; CVD Carbon-Coated Carbonized Loofah Sponge Loaded with a Directionally Arrayed MXene Aerogel for Electromagnetic Interference Shielding. *J. Mater. Chem. A* **2021**, *9*, 358-370.

(28) Han, M.; Yin, X.; Hantanasirisakul, K.; Li, X.; Iqbal, A.; Hatter, C. B.; Anasori, B.; Koo, C. M.; Torita, T.; Soda, Y.; Zhang, L.; Cheng, L.; Gogotsi, Y.; Anisotropic MXene Aerogels with a Mechanically Tunable Ratio of Electromagnetic Wave Reflection to Absorption. *Adv. Opt. Mater.* **2019**, *7*, 1900267.

(29) Yang, G. Y.; Wang, S. Z.; Sun, H. T.; Yao, X. M.; Li, C. B.; Li, Y. J.; Jiang, J. J.; Ultralight, Conductive $Ti_3C_2T_x$ MXene/PEDOT:PSS Hybrid Aerogels for Electromagnetic Interference Shielding Dominated by the Absorption Mechanism. *ACS Appl. Mater. Interfaces* **2021**, *13*, 57521-57531.

(30) Zhu, G.; Giraldo Isaza, L.; Huang, B.; Dufresne, A.; Multifunctional Nano-cellulose/Carbon Nanotube Composite Aerogels for High-Efficiency Electromagnetic Interference Shielding. *ACS Sustai. Chem. Eng.* **2022**, *10*, 2397-2408.

(31) Hu, P.; Lyu, J.; Fu, C.; Gong, W. B.; Liao, J.; Lu, W.; Chen, Y.; Zhang, X.; Multifunctional Aramid Nanofiber/Carbon Nanotube Hybrid Aerogel Films. *ACS Nano* **2020**, *14*, 688-697.

(32) Huang, H; Liu, C; Zhou, D.; Jiang, X.; Zhong, G; Yan, D.; Li, Z.; Cellulose Composite Aerogel for Highly Efficient Electromagnetic Interference Shielding. *J. Mater. Chem. A*

2015, *3*, 4983-4991.

(33) Puthiyedath Narayanan, A.; Narayanan Unni, K. N.; Peethambharan Surendran, K.; Aerogels of V_2O_5 Nanowires Reinforced by Polyaniline for Electromagnetic Interference Shielding. *Chem. Eng. J.* **2021**, *408*, 127239.

(34) Zeng, Z.; Wu, T.; Han, D.; Ren, Q.; Siqueira, G.; Nystrom, G.; Ultralight, Flexible, and Biomimetic Nanocellulose/Silver Nanowire Aerogels for Electromagnetic Interference Shielding. *ACS Nano* **2020**, *14*, 2927-2938.
